# Mechanical movements generated by movable lipids break endosomal barriers for enhanced mRNA therapeutics

**DOI:** 10.1126/sciadv.aef1695

**Published:** 2026-07-01

**Authors:** Zilu Li, Jie Qin, Jiayu Zhang, Yumiao Chen, Daohan Yu, Yiran Zhang, Shengfei Yang, Zhuoting Li, Juanjuan Zheng, Jinquan Cai, Fan Huang, Jianqing Gao, Yu Zhao

**Affiliations:** ^1^ State Key Laboratory of Advanced Drug Delivery and Release Systems, Institute of Pharmaceutics, School of Pharmacy, Zhejiang University, Hangzhou 310058, China.; ^2^ Department of Neurosurgery, The Second Affiliated Hospital of Harbin Medical University, Harbin 150086, China.; ^3^ State Key Laboratory of Advanced Medicals and Devices, Tianjin Key Laboratory of Radiation Medicine and Molecular Nuclear Medicine, Tianjin Institutes of Health Science, Institute of Radiation Medicine, Chinese Academy of Medical Sciences & Peking Union Medical College, Tianjin 300192, China.; ^4^ State Key Laboratory of Reproductive Medicine and Offspring Health, Center for Reproductive Medicine, Institute of Women, Children and Reproductive Health, Shandong University, Jinan 250012, China.; ^5^ Zhejiang Key Laboratory of Preclinical Research on Antitumor Drugs, School of Pharmacy, Zhejiang University, Hangzhou 310058, China.; ^6^ Eye Center, The Second Affiliated Hospital, School of Medicine, Zhejiang University, Hangzhou, China.

## Abstract

Lipid nanoparticles (LNPs), composed of ionizable lipids, phospholipids, cholesterol, and PEGylated lipids, have been successfully used in messenger RNA (mRNA) vaccine development. Despite substantial progress, endosomal entrapment after cellular internalization is still a critical bottleneck limiting the vaccine efficacy. While the efforts to optimize lipid p*K*_a_, spatial conformation, and LNP composition have been made, further fine-tuning of these parameters shows diminishing returns in improving effectiveness. Here, we propose a previously unreported parameter, programmable mechanical movement. LNPs are expected to destabilize endosomal membranes through conducting mechanical movements under specific inputs, achieving robust endosomal escape. Specifically, we demonstrate a light-emitting diode (LED)–driven movable lipid (i.e., phenylazothiazole lipid) capable of performing mechanical movements. We integrate the movable lipids into the BNT162b2 formulation from Pfizer-BioNTech. Upon LED irradiation, the movable lipids within LNPs function as molecular rotors, thereby facilitating endosomal membrane destabilization. This strategy has achieved exciting preclinical results in enhancing mRNA-LNP cancer vaccine efficacy.

## Teaser

LED-driven movable lipids function as molecular rotors to facilitate endosomal membrane disruption for enhanced mRNA therapeutics.

## INTRODUCTION

Lipid nanoparticles (LNPs) have served as highly effective delivery platforms for mRNA (*1*–*6*), as demonstrated by the successful development of COVID-19 vaccines, including BNT162b2 (Pfizer-BioNTech) (*7*) and mRNA-1273 (Moderna) (*8*). Despite substantial progress, endosomal entrapment continues to be a critical bottleneck limiting the efficacy of LNP-based mRNA vaccines (*9*–*11*). Fewer than 2% of delivered mRNA achieves endosomal escape and reaches the cytoplasm to enable translation (*12*–*16*). This substantial loss of payloads typically necessitates the administration of higher mRNA doses to attain therapeutic outcome, which is not considered a scientifically optimal approach. Numerous efforts have been made to optimize the p*K*_a_ or spatial conformation of lipids, and the composition of LNPs for enhancing endosomal escape efficiency (*17*–*22*). However, fine-tuning these routine parameters appears to have reached a ceiling in further efficacy improvement.

Molecular machines, such as dynein, kinesin, and their relatives, enable precise regulation of intracellular, transmembrane, and intercellular transport of biomacromolecules through executing rotational and translational movements at the cellular level (*23*–*25*). Specifically, these molecular machines are able to execute specific mechanical movements (outputs) by undergoing conformational changes in response to appropriate external stimuli (inputs) (*26*). Inspired by this, we propose to transcend current LNP design paradigms by introducing a previously unreported parameter, programmable mechanical movement. After cellular entry via endocytosis, LNPs are expected to destabilize endosomal membranes through conducting mechanical movements under specific inputs, achieving robust transmembrane transport to the cytoplasm (i.e., endosomal escape) in a manner analogous to molecular machines.

**Fig. 1.**
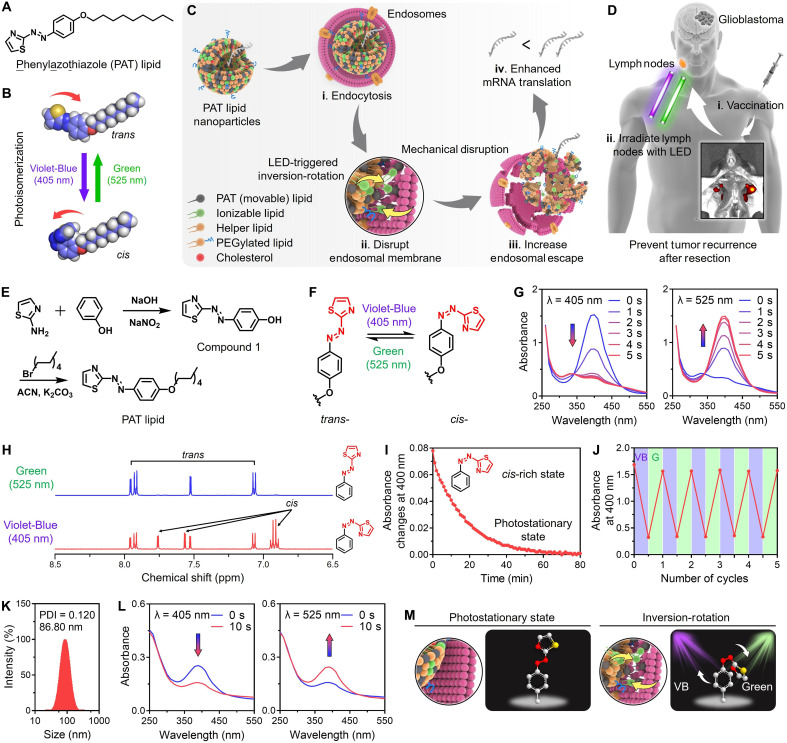
LED-driven mechanical movements generated by PAT LNPs break endosomal barriers. (**A**) Chemical structure of PAT lipid. (**B**) Cartoon illustration of LED-induced reversible isomerization of PAT lipids. (**C**) Schematic illustration of the potential mechanism of breaking endosomal barriers through LED-driven mechanical movements. (i) PAT LNPs enter the APCs via endocytosis; (ii) reversible isomerization of Azo units triggered by programmed LED irradiation causes PAT lipids to undergo continuous inversion-rotation, thereby destabilizing the endosomal membranes; (iii) PAT LNPs increase endosomal escape efficiency, and (iv) enhance mRNA translation. (**D**) Vaccination with PAT LNP–based vaccines, followed by localized and programmed LED irradiation of the draining LNs prevents postoperative recurrence of GBM. (**E**) Synthesis route of PAT lipid. (**F**) PAT lipids reversibly convert between trans- and cis-isomers upon exposure to two different LED wavelengths. (**G**) UV-Vis absorption spectrum of PAT lipids before and after irradiation with violet-blue light (405 nm, 5 s) and green light (525 nm, 5 s) in acetonitrile, respectively. (**H**) ^1^H NMR spectrum of Azo units after irradiation with different LED light. (**I**) Thermal relaxation of cis-isomers of PAT lipids in acetonitrile. After irradiation with violet-blue light for 1 min, the light source was removed, and the UV-Vis absorbance at 400 nm was recorded in the dark. (**J**) Repeated irradiation with alternating wavelengths led to reversible changes in absorbance at 400 nm. (**K**) Diameter distributions of PAT LNPs measured by DLS, together with the PDI value. (**L**) UV-Vis absorption spectrum of PAT LNPs in Hepes buffer at 37°C after irradiation with different LED light (10 s). (**M**) Schematic illustration of continuous inversion-rotation of PAT lipids caused by programmed LED irradiation.

Visible light-emitting diode (LED) light represents an attractive external stimulus due to its noninvasiveness, appropriate tissue penetrability, and precise spatiotemporal resolution (*27*–*34*). Herein, we designed a LED-driven movable lipid capable of performing mechanical movements, and integrated it into the traditional four-component LNP framework as the fifth component (Fig. 1A). The phenylazothiazole (PAT) serves as the key structural moiety of the movable lipid (denoted as PAT lipid). PAT lipid exhibits reversible photoisomerization between an extended trans-configuration and a compact cis-configuration under alternating violet-blue (405 nm) and green (525 nm) light irradiation, which induces continuous rotation-inversion dynamics, accompanied by stretch-shrink movements (Fig. 1B). Building upon the BNT162b2 from Pfizer-BioNTech, we developed a previously unreported LNP formulation, composed of PAT movable lipid, ALC-0315 ionizable lipid, PEGylated lipid, 1,2-distearoyl-sn-glycero-3-phosphocholine (DSPC) helper lipid, and cholesterol (denoted as PAT LNP). After entering the cells, PAT LNPs electrostatically adhere to the negatively charged endosomal membranes. Upon programmed LED irradiation, the PAT lipids within LNPs function as molecular rotors, thereby facilitating endosomal membrane destabilization and disruption through mechanical movements (Fig. 1C). Our study revealed that, under LED irradiation, PAT LNPs significantly enhanced mRNA translation both in vitro and in vivo compared to commercial BNT162b2 LNPs.

As a proof of concept, we evaluated the potential of PAT LNPs as the mRNA vaccine carrier for cancer intervention. Our results showed that the incorporation of PAT lipids did not compromise the lymph node (LN) accumulation capability of the original four-component LNP formulation following subcutaneous administration. Cell-type analysis revealed that mRNA translation occurred primarily in antigen-presenting cells (APCs), including dendritic cells (DCs) and macrophages. Programmed LED irradiation of LNs nearly doubled mRNA expression levels, demonstrating strong potential to elicit a robust antitumor immune response. As a result, the PAT LNP–based vaccination strategy significantly enhanced antigen-specific cytotoxic T cell activation and exhibited potent efficacy in suppressing tumor growth, reducing recurrence, and inhibiting lung metastasis in a mouse melanoma model. Glioblastoma (GBM) is the most lethal form of central nervous system cancer, partly due to the inability to combat its postoperative recurrence (*35*, *36*). To induce immune activation in the head region, mice were vaccinated with the PAT LNP–based vaccines in the interscapular region and received programmed LED irradiation targeting the axillary LNs (*37*–*39*). As expected, effective prevention of GBM recurrence was observed in the intracranial region following surgical resection (Fig. 1D). Collectively, we present a rational design paradigm for LNPs through the incorporation of movable lipids into a traditional four-component system. The movable lipid demonstrated here was designed to perform mechanical movements through the consumption of photons, thereby allowing for efficient transport of mRNA from endosomes to the cytoplasm. Our study represents a groundbreaking and bold endeavor in the field of LNPs, offering insights for the rational design of next-generation mRNA delivery systems.

## RESULTS

### Characterization of PAT movable lipid and PAT LNP

We synthesized PAT lipid and confirmed its chemical structure (Fig. 1E and fig. S1). Subsequently, we characterized its photoisomerization kinetics via UV-Vis absorption spectroscopy (Fig. 1F). As shown in Fig. 1G, irradiation with violet-blue light for 5 s gradually reduced the absorption peak corresponding to the π-π* transition of the trans-PAT lipids, suggesting the formation of a photostationary state dominated by the cis-configuration. This spectral change was rapidly reversed after 5 s of green light exposure, demonstrating highly efficient and reversible photoisomerization. To confirm the dynamic isomerization of the PAT lipid, we acquired its ^1^H nuclear magnetic resonance (^1^H NMR) spectra immediately after 30 s of irradiation (sufficient to reach the photostationary state) with violet-blue and green light, respectively. The aromatic protons of the two PAT lipid isomers exhibit distinct chemical shifts, the trans-form is 7.1 to 8.0 parts per million (ppm), and the cis-form is 6.8 to 7.8 ppm. According to the results, irradiation with violet-blue light shifted the equilibrium toward the cis-configuration, while green light promoted the trans-configuration (Fig. 1H). NMR analysis following violet-blue light exposure showed a 30 to 40% residual trans-PAT lipid population, which was inconsistent with the results of UV-Vis absorption spectroscopy. We attributed this to the short thermal relaxation time of the cis-configuration, which allowed partial reversion to the trans-form before or during spectral acquisition. This hypothesis is corroborated by thermal relaxation studies, wherein approximately 35% of the cis-PAT lipids reverted within 10 min postirradiation (Fig. 1I). Furthermore, the PAT lipids demonstrated rapid reversible switching over multiple cycles of alternating violet-blue and green light, providing a structural basis for mechanical movements (Fig. 1J). Then, we incorporated the PAT lipids into clinically available BNT162b2 LNP formulation. We first determined the maximum tolerable PAT lipid ratio by formulating LNPs with increasing percentages (10 to 50%) of the PAT lipids. As shown in Fig. 1K and fig. S2A, incorporating more than 30% PAT lipid resulted in a significant increase in particle size. Moreover, after 24-hour incubation at 37°C, these high-PAT lipid formulations exhibited approximately 5-fold size enhancement and a substantially increased polydispersity index (PDI), indicating poor colloidal stability (fig. S2B). Therefore, a formulation containing 20% PAT lipids was used to achieve an optimal balance between maximizing PAT lipid content and maintaining acceptable stability (fig. S2, C to E). This formulation also exhibited an encapsulation efficiency for mRNA comparable to that of the BNT162b2 LNP, hence named PAT LNP (fig. S2F). Transmission electron microscopy (TEM) image exhibited a spherical morphology with a solid core (fig. S3A).

The photoisomerization of PAT lipids within PAT LNPs was then evaluated in Hepes buffer (20 mM, pH 7.2) at 37°C. As shown in Fig. 1L and fig. S3B, 10 s of irradiation with violet-blue or green light resulted in cis-rich or trans-rich photostationary states, respectively. The slightly slower isomerization of PAT lipids in LNPs, compared to free PAT lipids, might be attributed to the fact that these lipids are in the tightly packed solid nanoparticles. The reversible isomerization of PAT lipids within LNPs was evaluated under multiple cycles of alternating violet-blue and green light irradiation (10 s for each). As expected, the PAT lipid switched rapidly and reversibly between their cis and trans states (fig. S3C), which induces pronounced changes in its length, width, and height. Thus, the PAT lipids can perform stretch-shrink motions, showing the potential to facilitate endosomal membrane disruption during the escape (Fig. 1M). Moreover, once irradiation ceased, the thermal relaxation was completed within approximately 2 min, allowing for precise control of the LED-driven mechanical movements (fig. S3D). We used alternating irradiation with blue-violet and green LED light to ensure that PAT lipids can reversibly and continuously convert between trans- and cis-isomers, thereby generating mechanical movement to destabilize the endosomal membrane. Specifically, LED irradiation at the two wavelengths was alternated every 10 s for in vitro experiments and every 15 s for animal studies. Notably, these single-wavelength irradiation intervals are much shorter than 2 min. Therefore, before significant thermal relaxation occurs, the next cycle of photoisomerization is already initiated by the alternating light source.

### Mechanical movements facilitate endosomal escape

**Fig. 2.**
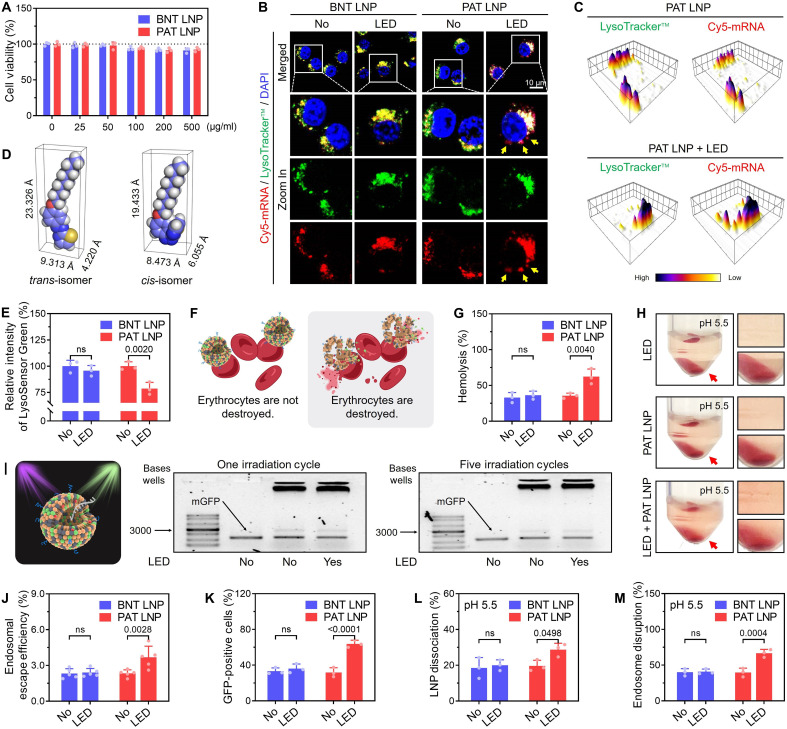
Mechanical movements facilitate endosomal escape. (**A**) Cell viabilities of DC2.4 cells after 24 hours of incubation with various concentrations of BNT LNPs and PAT LNPs, respectively. (**B**) Representative fluorescence images of the endosomal escape of Cy5-labeled mRNA-loaded different LNPs in DC2.4 cells, respectively. After 1.5 hours of incubation with LNPs, the cells were irradiated with LED light for 10 min. LED irradiation at two different wavelengths was alternated every 10 s. After another 20-min incubation, LysoTracker Green was added. Scale bar, 10 μm. (**C**) Representative surface plot of quantification of endosomes (LysoTracker, left) and Cy5-mRNA (red, right) within cells. (**D**) Molecular dimensions of trans-isomer and cis-isomer of PAT lipid. (**E**) Fluorescence intensity of LysoSensor Green in DC2.4 cells after incubation with different LNPs, with or without LED irradiation. (**F** to **H**) Hemolysis assay to evaluate the membrane disruption by PAT LNPs at pH 5.5, with or without LED irradiation. (**I**) Representative agarose gel electrophoresis result of mGFP-loaded PAT LNPs after one (left) and five (right) irradiation cycles. (**J**) Quantification of the endosomal escape efficiency. (**K**) mGFP (1 μg/ml) transfection efficacy of different LNPs, with or without LED irradiation, tested on DC2.4 cells by quantifying GFP-positive cells. (**L**) PAT LNP dissociation determined by a FRET assay after mixing with endosomal mimics at pH 5.5, with or without LED irradiation. The endosomal mimics were prepared by mixing DOPS, DOPC, and DOPE (25/25/50, mol/mol), and labeled by rhodamine-phosphatidylethanolamine (Rhod-PE) and *N*-(7-nitrobenzo-2-oxa-1,3-diazole-phosphatidylethanolamine (NBD-PE). (**M**) Endosome disruption determined by a FRET assay after mixing with PAT LNPs at pH 5.5. Data are presented as mean ± SD from *n* biologically independent samples (*n* = 3). Statistical significance was analyzed by two-way ANOVA with Sidak’s multiple comparisons test.

Then, we explored the potential of the mechanical movements generated by PAT lipids for facilitating endosomal escape in APCs. The cytotoxicity of PAT LNPs was first examined in DC2.4 cells. BNT LNPs were selected as a benchmark for comparison. As shown in Fig. 2A, similar cell viability was observed for both PAT LNPs and BNT162b2 LNPs (denoted as BNT LNPs) after 24 hours of incubation, even at a high concentration of 500 μg/ml. This result indicated that the integration of PAT lipids does not compromise the biocompatibility of the established clinical formulation. Because the endosomal escape efficiency can also be influenced by the internalization pathway, we therefore studied the mechanisms of the LNPs with specific inhibitors against clathrin-mediated endocytosis (chlorpromazine) (*40*), caveolae-mediated endocytosis (nystatin) (*41*), and macropinocytosis (amiloride) (*42*). The internalization mechanisms were similar across the tested LNPs. Both PAT LNPs and BNT LNPs entered cells primarily through clathrin-mediated and caveolae-mediated endocytosis, rather than macropinocytosis (fig. S4A).

The endosomal escape of the LNPs was visualized by confocal laser scanning microscopy. Briefly, Cy5-labeled mRNA-loaded LNPs were prepared and incubated with DC2.4 cells for 1.5 hours. The cells were then irradiated for 10 min with LED light, alternating every 10 s between two wavelengths (i.e., 405 and 525 nm). After a further 20-min incubation, the cells were stained with LysoTracker Green. As shown in Fig. 2B, LED irradiation triggered the efficient release of Cy5-labeled mRNA (red) carried by PAT LNPs from endosomes (green) into the cytoplasm, whereas in LED absence, the mRNA-PAT LNPs remained largely trapped within endosomes. In contrast, almost all mRNA-BNT LNPs were trapped in the endosome at this time point, regardless of LED irradiation. Further evidence for the LED-induced endosomal escape of mRNA-PAT LNPs came from the analysis of the red and green fluorescence surface plots (Fig. 2C). We further evaluated endosomal escape by calculating the Manders’ overlap coefficient (MOC) (*43*). The MOC value was calculated as the ratio of the red fluorescence signal intensity that colocalized with the green signal to the total red signal intensity. A lower MOC value indicates a higher endosomal escape efficiency. According to the results, LED irradiation led to significantly decrease the MOC value in PAT LNP group. The Pearson’s correlation scatter plot revealed a distribution of points below the diagonal and near the red fluorescence axis, indicating that a large amount of red fluorescent mRNA has been no longer within the green fluorescent endosomes (fig. S4B).

We then investigated the molecular dimensions of the PAT lipid in its cis- and trans-conformations (Fig. 2D). The results revealed pronounced alterations in its height, length, and width (*44*). These substantial dimensional changes can generate mechanical movements, which we attribute to the enhanced endosomal disruption responsible for the efficient escape. The disruption of endosomes was evaluated using the LysoSensor Green assay. LysoSensor probes are fluorescent in acidic endosomes but nonfluorescent at neutral pH. Consequently, the disruption of endosomes and the subsequent pH increase lead to a suppression of the fluorescence signal. Compared to cells treated with BNT LNPs, those in the PAT LNP group exhibited a significantly reduced fluorescence intensity following LED irradiation (Fig. 2E). We then used a hemolysis assay to evaluate the endosomolytic activity of the LNPs, a surrogate model that quantifies hemoglobin release upon exposure to red blood cells (RBCs) (*45*, *46*). The endosomolytic activity at pH 5.5 models the ability of LNPs to escape from endosomes (Fig. 2F). As expected, PAT LNPs demonstrated enhanced hemolytic activity under LED irradiation at this pH, compared to BNT LNPs (Fig. 2G). Direct visual observation of the samples also revealed a marked difference. Following LED irradiation, the supernatant in PAT LNP group exhibited noticeable turbidity and a pale red hue, indicating the presence of erythrocyte fragments and the release of hemoglobin (Fig. 2H). To further quantify the endosomal escape efficiencies, we used a Split Luciferase Endosomal Escape Quantification (SLEEQ) assay (*47*, *48*). According to the results, the efficiency of the PAT LNP group increased 1.6-fold from 2.3 to 3.7% upon LED irradiation, whereas the BNT LNP group showed a negligible increase (Fig. 2J).

Next, we selected green fluorescent protein mRNA (mGFP) as a model cargo to evaluate how LED irradiation of PAT LNPs affects mRNA transfection efficiency in DC2.4 cells. As shown in Fig. 2K, LED irradiation resulted in a 2-fold increase in transfection efficiency in the PAT LNP group. However, negligible differences were observed in the BNT LNP group with or without irradiation (fig. S4C). Then, fresh single-layer skin harvested from mouse inguinal regions was placed directly above the LED light source to serve as a modified irradiation setup. DC2.4 cells incubated with mGFP-loaded PAT LNPs were then irradiated with this LED source. As expected, GFP expression was significantly enhanced after LED irradiation, indicating effective skin penetration of the LED light for driving PAT LNPs (fig. S4, D and E). Because both PAT and BNT LNPs exhibit similar encapsulation efficiency and cellular uptake, the enhanced GFP expression can be attributed to the improved efficiency of PAT LNPs in delivering mGFP to the cytoplasm following LED irradiation (fig. S4F). Moreover, mRNA-PAT LNPs exhibited minimal mRNA leakage even after five LED irradiation cycles, as confirmed by agarose gel electrophoresis (Fig. 2I and fig. S4G). We speculate that effective light-induced LNP dissociation occurred only upon concurrent contact with the endosomal membranes. Specifically, although the mechanical movements generated by PAT lipids is not sufficient to directly destabilize LNPs, it can promote lipid rearrangement during the escape process, thereby promoting the destruction of the endosomal membranes and LNP themselves. The liposome-based Förster resonance energy transfer (FRET) assays confirmed our hypothesis (*18*, *49*). As shown in Fig. 2 (L and M), LED irradiation triggered effective LNP dissociation and endosomal disruption in the PAT LNP group, in stark contrast to the minimal response observed in BNT LNPs. To further investigate the structure-activity relationship of the PAT lipid in enhancing mRNA expression, we synthesized a series of PAT lipid analogs with systematically varied tail lengths (C0, C2, C5, C9, and C13). Our results demonstrated a clear positive correlation: As the tail length increased, so did the endosomal escape efficiency of the corresponding LNPs and their resulting mRNA expression levels (fig. S5). These findings confirm the crucial role of the PAT lipid–induced mechanical movements.

### Mechanical movements enhance mRNA expression in LNs

**Fig. 3.**
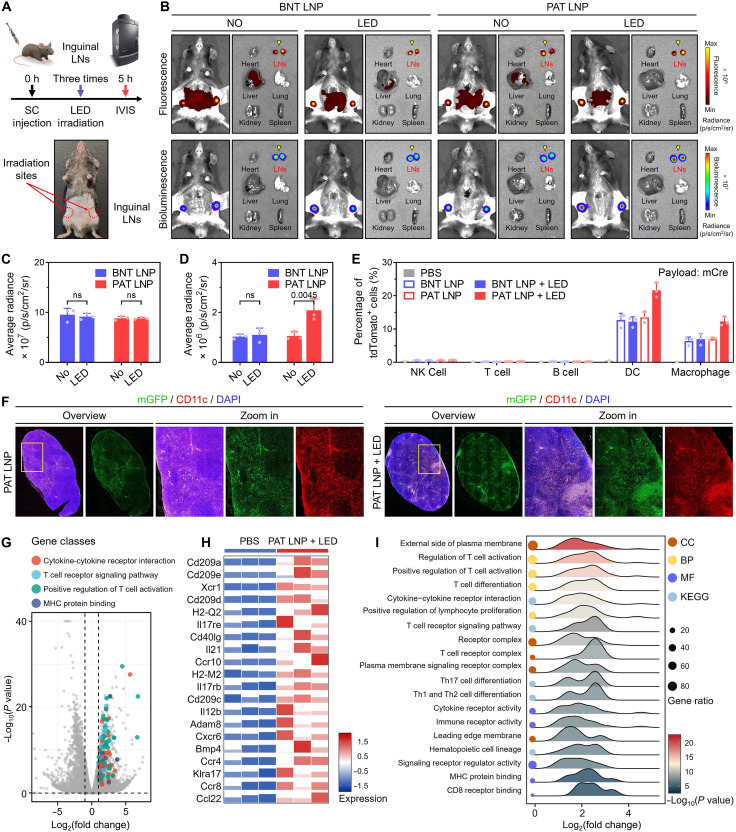
Mechanical movements generated by PAT LNPs enhance mRNA expression in LNs. (**A**) Schematic illustration of the experimental design. C57BL/6 mice were irradiated with LED light during the first 15 min of each of the second, third, and fourth hours after subcutaneous injection of different formulations. LED irradiation at two different wavelengths was alternated every 15 s. Images were taken at 5 hours postinjection. (**B**) Representative fluorescence (top) and bioluminescence (bottom) images of the mice after subcutaneous injection of DiR-labeled mLuc-loaded LNPs (mRNA, 5 μg per mouse) measured by the IVIS imaging system. Representative ex vivo fluorescence and bioluminescence images of inguinal LNs (indicated by yellow arrows) and main organs are also shown. (**C**) Quantitative analysis of the fluorescence intensity and (**D**) bioluminescence intensity within inguinal LNs. (**E**) Cre-mediated gene recombination in Ai14 mice. The percentage of tdTomato-positive (tdTomato^+^) cells across different immune cell populations following subcutaneous injection of mCre-loaded LNPs. (**F**) Fluorescent images of mGFP expression in the whole LNs. (**G**) Volcano plot depicted differentially expressed genes between the PBS control and treatment group from RNA sequencing analysis. The 127 highlighted up-regulated genes (denoted by distinct colors) correlated with key immune processes. (**H**) Heatmap showing the top 20 representative genes from the set of 127 genes. (**I**) GO and KEGG analysis of differentially expressed genes between the PBS control and treatment group. Data are presented as mean ± SD from *n* biologically independent samples (*n* = 3). Statistical significance was analyzed by two-way ANOVA with Sidak’s multiple comparisons test.

We further evaluated the impact of LED irradiation on the in vivo mRNA delivery efficiency of the PAT LNPs. The firefly luciferase mRNA (mLuc) was encapsulated within 1,1′-dioctadecyl-3,3,3′,3′-tetramethylindotricarbocyanine iodide (DiR)–labeled LNPs, enabling simultaneous tracking of both mRNA-LNP distribution and mRNA expression in the mouse model. Following the subcutaneous injection of different mLuc-LNPs, the inguinal LNs of C57BL/6 mice were exposed to LED irradiation during the first 15 min of each of the second, third, and fourth hours (i.e., 60 to 75, 120 to 135, and 180 to 195 min). The light source alternated between two wavelengths (i.e., 405 and 525 nm) every 15 s (*50*). Images were taken at 5 hours postinjection (Fig. 3A). BNT LNPs were used as the benchmark for comparison. As shown in Fig. 3 (B to D), while both PAT LNPs and BNT LNPs exhibited comparable LN accumulation (similar fluorescence intensity), LED irradiation significantly enhanced the bioluminescence signal (2-fold) only in the PAT LNP group, with a negligible effect observed in the BNT LNP group. We also investigated the relationship between irradiation duration/frequency and mRNA expression levels. According to the results, the levels of mRNA expression gradually increased with prolonged irradiation time or an increased number of irradiation cycles, confirming the key role of PAT lipids in facilitating mRNA expression (fig. S6, A and B).

To elicit potent antitumor immunity, a cancer vaccine carrier is required that enables efficient mRNA expression in APCs, leading to enhanced antigen cross-presentation. The specific types of immune cells targeted by PAT LNPs in the spleen were profiled next. In this study, we used genetically engineered Ai14 reporter mice, in which successful intracellular delivery of Cre recombinase mRNA (mCre) triggers the permanent expression of the red fluorescent protein tdTomato (fig. S6C) (*51*). We analyzed the percentage of tdTomato-positive cells by flow cytometry after intravenous injection of mCre-loaded LNPs. While both BNT LNPs and PAT LNPs effectively induced tdTomato expression in DCs and macrophages, the expression level mediated by PAT LNPs was markedly improved upon LED irradiation, underscoring its potential as a robust vaccine carrier (Fig. 3E and fig. S6D). Immunofluorescence analysis of LN sections revealed that mGFP-loaded PAT LNPs mediated efficient GFP (green) expression in DCs (red). Furthermore, this signal was significantly intensified upon LED irradiation (Fig. 3F and fig. S7A). Cytokine analysis at the injection site showed no significant increase in inflammatory response from PAT LNPs compared to the BNT LNPs, demonstrating that PAT LNP formulation is well tolerated (fig. S7B). To strengthen the generalizability of our strategy, we also incorporated the PAT lipids into SM102 LNP formulation (denoted as PAT-SM102 LNP) and evaluated the mRNA expression efficacy after LED irradiation. SM102 LNPs were used as the benchmark for comparison. PAT-SM102 LNPs and SM102 LNPs exhibited comparable LN accumulation. As expected, LED irradiation significantly enhanced the bioluminescence signal (2.9-fold) only in the PAT-SM102 LNP group, with a negligible effect observed in the SM102 LNP group (fig. S7, C to E). These results confirm the compatibility and generalizability of the PAT lipids.

To further elucidate the potential of movable lipid-based strategy in immune activation, we conducted RNA sequencing of LN tissues following treatment. The mRNA encoding the model antigen ovalbumin (mOVA) was used as a model cargo. The results revealed 1269 up-regulated and 1349 down-regulated genes, with 127 of the up-regulated genes mapped to key immune processes, including cytokine-cytokine receptor interaction, T cell receptor signaling pathway, positive regulation of T cell activation, and major histocompatibility complex (MHC) protein binding, in the treatment group (i.e., PAT LNP administration combined with LED irradiation) compared to the phosphate-buffered saline (PBS) group (Fig. 3G). The top 20 genes from these 127 genes were presented in the heatmap (Fig. 3H). GO (Gene Oncology) and KEGG (Kyoto Encyclopedia of Genes and Genomes) enrichment analysis revealed the up-regulated genes involved in T cell activation and differentiation, cytokine-cytokine receptor interaction, and receptor complex (Fig. 3I). These results collectively demonstrated that PAT LNP administration combined with LED irradiation induced robust immune activation.

### Antigen-specific immune activation and tumor suppression

**Fig. 4.**
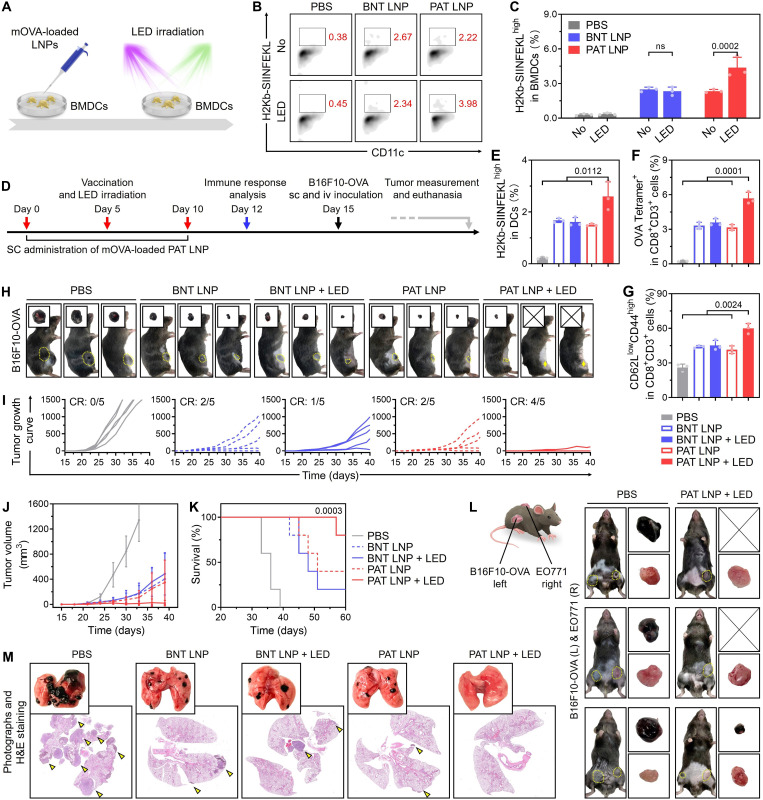
PAT LNP–based vaccines suppress melanoma growth and metastasis. (**A**) Schematic illustration of the experimental design. BMDCs were incubated with mOVA-loaded LNPs (mOVA, 1 μg/ml) for 3 hours, then received LED irradiation. (**B**) Representative flow cytometry analysis and (**C**) statistical analysis of the expression of H2kb-SIINFEKL in BMDCs. (**D**) Schematic illustration of the timeline for vaccination (mOVA, 15 μg per mouse) by different formulations, immune response analysis, and B16F10-OVA tumor inoculation (subcutaneous/intravenous injection) in the C57BL/6 mouse model. (**E**) Flow cytometry analysis of the population of DC cells that express H2kb-SIINFEKL (gated on Ly6G^−^CD11c^+^ cells) in inguinal LNs. (**F**) Flow cytometry analysis of the population of CD8^+^CD3^+^ T cells that bear T cell receptors binding to H2Kb OVA tetramer-SIINFEKL in inguinal LNs. (**G**) Flow cytometry analysis of the population CD62L^low^CD44^high^ T cells (gated on CD8^+^CD3^+^ cells) in inguinal LNs. (**H**) Photographs of B16F10-OVA tumors. (**I**) Individual tumor growth curves. CR, complete response. (**J**) Average tumor growth kinetics. (**K**) Survival of B16F10-OVA tumor–bearing mice. (**L**) Photographs of B16F10-OVA and EO771 tumors. For the rechallenge, B16F10-OVA tumor cells were subcutaneously injected at the left flank of mice, while EO771 tumor cells were inoculated into the opposite flank. (**M**) Photographs and H&E staining images of the lung tissues. Data are presented as mean ± SD from *n* biologically independent samples (C, E, F, and G, *n* = 3; J and K, *n* = 5). Statistical significance was analyzed by two-way ANOVA with Sidak’s multiple comparisons test for (C), one-way ANOVA with Tukey’s multiple comparisons test for (E) to (G), two-way ANOVA with Tukey’s test for (J), and log-rank (Mantel-Cox) test for (K).

The antigen cross-presentation was further studied in bone marrow–derived dendritic cells (BMDCs). The mOVA-loaded PAT LNPs were incubated with BMDCs and received LED irradiation (Fig. 4A). The cross-presentation of OVA in BMDCs was analyzed by flow cytometry via the detection of H2kb-SIINFEKL complexes. As shown in Fig. 4 (B and C), LED irradiation induced a 1.9-fold enhancement in the cross-presentation of OVA peptides on MHC-I in BMDCs treated with mOVA-loaded PAT LNPs, relative to nonirradiated controls. Conversely, no significant increase in antigen cross-presentation was induced by LED irradiation in BMDCs treated with BNT LNPs. These results demonstrated that the key role of LED light-mediated mRNA cytoplasmic transport by PAT LNPs in enhancing antigen cross-presentation also showed the potential of the movable lipid-based strategy for initiating T cell–dependent antitumor immunity in vivo.

To compare the ability of PAT LNP– and BNT LNP–based vaccines to induce antigen-specific cytotoxic T cell–mediated immune responses, mice were subcutaneously vaccinated with 15 μg of mOVA per mouse and received LED irradiation. Briefly, mice received a prime vaccination on day 0 with booster doses on days 5 and 10 (Fig. 4D). LNs were then collected on day 12 to assess cross-presentation and OVA-specific CD8^+^ T cell activation after treatments. As expected, mice vaccinated with PAT LNP formulation and exposed to LED irradiation showed significantly improved cross-presentation efficiency (H2kb-SIINFEKL^high^, 2.51%) in DCs compared to those receiving only vaccination without irradiation (1.55%). In the BNT LNP group of mice, LED irradiation did not improve cross-presentation efficiency (NO versus LED, 1.76% versus 1.50%) (Fig. 4E and fig. S8A). OVA-specific CD8^+^ T cell activation was evaluated using a tetramer assay. As demonstrated in Fig. 4F and fig. S8B, a negligible population of OVA-specific CD8^+^ T cells was detected in the PBS control group (OVA Tetramer^+^, 0.23%). Vaccination with LNP-based formulations elicited robust OVA-specific T cell responses (BNT LNP, 3.28%; PAT LNP, 2.92%). Notably, LED irradiation only increased the OVA-specific CD8^+^ T cell population only in the PAT LNP group (5.46%), highlighting the critical role of LED light-mediated mRNA cytoplasmic transport by PAT LNPs in promoting antigen-specific cytotoxic T cell responses. Furthermore, the administration of PAT LNP–based vaccines combined with LED irradiation resulted in the significant elevation in the populations of CD62L^low^CD44^high^ effector memory T cells (T_EM_, 60.5%) and CD62L^high^CD44^high^ central memory T cells (T_CM_, 20.2%) in the LNs (Fig. 4G and fig. S8C), which are critical for long-term immunity and tumor metastasis surveillance.

To evaluate the prophylactic efficacy, mice were challenged with OVA-expressing B16F10 murine melanoma (B16F10-OVA) tumor cells on day 15. As shown in Fig. 4 (H to K), the tumors in the unvaccinated control group (treated by PBS) grew rapidly until reaching the humane endpoint. Excitingly, the administration of PAT LNP–based vaccines combined with LED irradiation achieved a 67% (complete response, 4/6) tumor-free rate and prolonged survival time of the mice. This efficacy was superior to all other experimental groups. Similar therapeutic tendencies were observed in the tumor slices by hematoxylin and eosin (H&E) staining (fig. S8D). T cell–dependent immune responses primed by DCs are antigen specific. To further confirm antigen-specific cytotoxic T cell responses, the vaccinated and irradiated mice were subcutaneously inoculated with B16F10-OVA tumor cells in the left flank and noncognate EO771 murine mammary tumor cells in the right flank. Normal mice without any treatments were inoculated with these two tumor cells, which were used as the control group. As expected, the B16F10-OVA tumor was completely inhibited in two out of three mice after treatment with the PAT LNP–based vaccines and LED irradiation. However, only slight suppression of EO771 tumor growth was observed (Fig. 4L). The selective inhibition of B16F10-OVA tumor growth was attributed to the infiltration and activation of OVA-specific cytotoxic T cells.

Next, after vaccination with different LNP formulations and LED irradiation, pulmonary metastasis of B16F10-OVA tumor cells was assessed following intravenous inoculation via the tail vein (Fig. 4D). On day 35, all mice were euthanized, and lung tissues were collected. As demonstrated in Fig. 4M, the lungs of PBS control mice exhibited extensive black tumor nodules. BNT LNP vaccination moderately reduced this pulmonary metastasis. The addition of LED irradiation provided minimal positive effect on the inhibitory efficacy. Although the nodules were still observed with PAT LNP treatment alone, its combination with LED irradiation completely inhibited tumor pulmonary metastasis. Histological analysis of H&E-stained lung sections corroborated these findings, with tumor nodules demarcated by black dotted lines (fig. S9).

### PAT LNP–based vaccines prevent postoperative recurrence of GBM

**Fig. 5.**
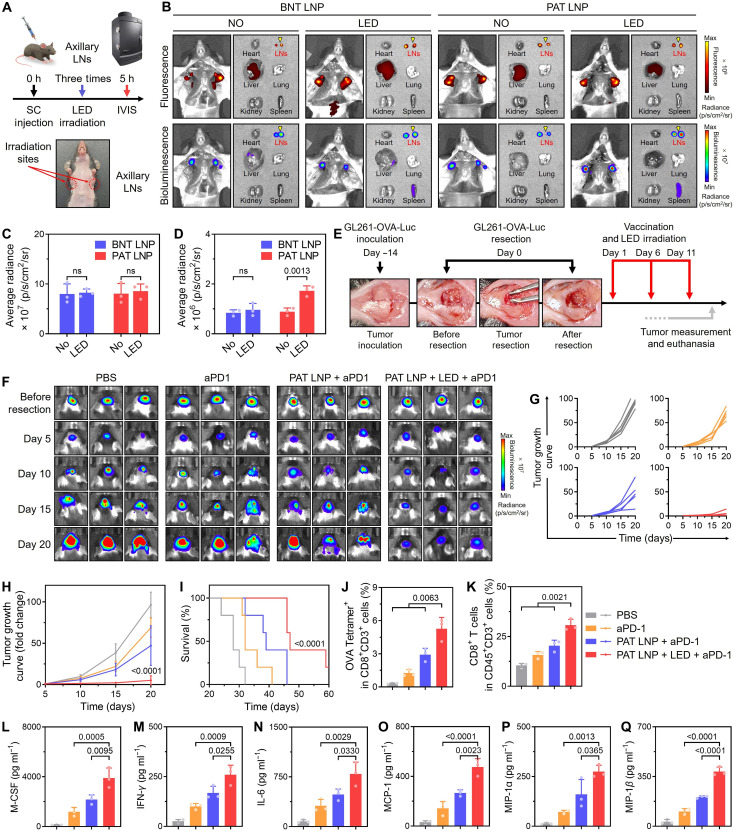
PAT LNP–based vaccines prevent postoperative recurrence of glioblastoma. (**A**) Schematic illustration of the experimental design. (**B**) Representative fluorescence (top) and bioluminescence (bottom) images of the mice after subcutaneous injection of DiR-labeled mLuc-loaded LNPs (mRNA, 5 μg per mouse) measured by the IVIS imaging system. Representative ex vivo fluorescence and bioluminescence images of axillary LNs (indicated by yellow arrows) and main organs are also shown. (**C**) Quantitative analysis of the fluorescence intensity and (**D**) bioluminescence intensity within inguinal LNs. (**E**) Schematic illustration of the timeline for GL261-OVA-Luc tumor inoculation, tumor resection, and vaccination (mOVA, 15 μg per mouse) by different formulations in C57BL/6 mouse model. (**F**) In vivo bioluminescence imaging of GL261-OVA-Luc tumor growth. (**G**) Individual tumor growth curves. (**H**) Average tumor growth kinetics. (**I**) Survival of GL261-OVA-Luc tumor–bearing mice. (**J**) Flow cytometry analysis of the population of CD8^+^CD3^+^ T cells that bear T cell receptors binding to H2Kb OVA tetramer-SIINFEKL in axillary LNs. (**K**) Flow cytometry analysis of the population of CD8^+^ T cells (gated on CD3^+^CD45^+^ cells) in GL261-OVA-Luc tumors. (**L** to **Q**) The levels of cytokines and chemokines in the blood. Data are presented as mean ± SD from *n* biologically independent samples (C, D, J, and K, *n* = 3; H and I, *n* = 5). Statistical significance was analyzed by two-way ANOVA with Sidak’s multiple comparisons test for (C) and (D), two-way ANOVA with Tukey’s test for (H), log-rank (Mantel-Cox) test for (I), and one-way ANOVA with Tukey’s multiple comparisons test for (J) and (K).

Recurrent GBM poses a critical clinical challenge following initial resection, with the vast majority of patients experiencing tumor progression (*52*). As recurrence is the primary cause of morbidity and mortality, developing effective therapeutic strategies for this disease stage remains a fundamental goal in neuro-oncology. Immune activation offers a critical benefit in preventing postsurgical GBM recurrence by reversing local immunosuppression (*53*). It promotes a robust antitumor response, enabling immune cells to target and eliminate residual tumor cells that survive resection. To evaluate the potential of our movable lipid-based strategy in preventing GBM recurrence, we sought to achieve robust immune activation in the head and neck region by ensuring efficient antigen expression in the axillary LNs. To test this, mice were subcutaneously injected with mLuc-loaded, DiR-labeled PAT LNPs in the interscapular region. Following injection, the axillary LNs were subjected to LED irradiation at two alternating wavelengths for 15 min during the second, third, and fourth hours (Fig. 5A). Bioluminescence imaging at 5 hours postinjection revealed that while both PAT LNPs and BNT LNPs accumulated comparably in the LNs, LED irradiation induced a significant 1.9-fold increase in signal exclusively in the PAT LNP group, with a negligible effect on BNT LNPs (Fig. 5, B to D). This indicates that the combination of PAT LNP–based vaccines with LED irradiation can induce efficient antigen expression in axillary LNs.

A GBM surgical resection model was then constructed. First, an orthotopic GBM mouse model was established by intracranial implantation of GL261 cells coexpressing OVA and Luc (denoted as GL261-OVA-Luc), with successful model establishment confirmed by detecting intracranial Luc expression using the In Vivo Imaging System (IVIS) imaging system. Subsequently, we surgically removed the tumor tissues (Fig. 5E). The GBM-bearing mice were able to tolerate the operative procedure with a low mortality rate (<10%) and an uneventful recovery without any signs of neurological impairment. We used the IVIS imaging system to assess the efficiency of surgical tumor resection by detecting the bioluminescence signals in the brains before and after surgery. Quantitative analysis of bioluminescence signals within defined regions of interest confirmed that the tumor resection efficiency exceeded 80%. We designated the day of tumor resection as day 0. Mice then received the PAT LNP–based vaccines with LED irradiation on days 1, 6, and 11. We systematically evaluated the antitumor efficacy of our strategy and immune checkpoint inhibitor anti–PD-1 antibody (aPD-1). As demonstrated in Fig. 5 (F to H), PAT LNP–based vaccine treatment improved the efficacy of immune checkpoint inhibitor therapy. When mice received LED irradiation, tumor growth was further suppressed and the survival rate of mice was significantly prolonged (Fig. 5I). The efficient antitumor effect is attributed to the robust cytotoxic T cell–mediated immune activation, which was first confirmed by a tetramer assay. According to the result, the combination therapy, with the addition of LED irradiation, induced the most potent OVA-specific CD8^+^ T cell response in the axillary LNs (OVA Tetramer^+^, 5.69%; Fig. 5J and fig. S10A). This robust immunity correlated with a significantly elevated infiltration of both CD8^+^ and CD4^+^ T cells into the brain tissue (CD8^+^, 29.5%; CD4^+^, 33.1%; Fig. 5K and figs. S10, B and C, and S11).

In addition, the activation of innate immunity was also studied by assessing the levels of cytokines and chemokines in the blood. As shown in Fig. 5 (L to Q), the combination therapy, with the addition of LED irradiation, elevated the levels of proinflammatory cytokines [including macrophage colony-stimulating factor, interferon-γ, and interleukin-6 (IL-6)] and chemokines [including monocyte chemoattractant protein-1, macrophage inflammatory protein-1α (MIP-1α), and macrophage inflammatory protein-1β (MIP-1β)]. IL-6 plays an important role in the proliferation and differentiation of cytotoxic T cells. These results confirmed that PAT LNPs represent a promising carrier for mRNA vaccines, capable of eliciting robust T cell–dependent adaptive immunity.

## DISCUSSION

Inspired by intracellular molecular machines, we developed a PAT movable lipid that can perform mechanical movements by consuming photons. By integrating clinically established LNP formulations with the PAT lipid as a fifth component, we developed a platform in which LED irradiation significantly enhanced mRNA delivery efficiency. This improvement is primarily attributed to the LED-driven endosomal membrane-disrupting capability of the PAT lipids, which potently promotes endosomal escape. Unlike traditional four-component LNPs, the PAT lipid–integrated LNP system operates through an additional, molecular machine-like mechanism that actively enhances cytosolic mRNA transport, representing an advance beyond the current LNP paradigm. Specifically, PAT lipids undergo reversible photoisomerization between an extended trans-configuration and a compact cis-configuration under alternating violet-blue and green light irradiation. This light-triggered isomerization induces continuous rotation-inversion dynamics accompanied by stretch-shrink movements at the molecular level, which generate sufficient mechanical strain to disrupt the structural stability of the endosomal membrane and promote effective endosomal escape. This strategic innovation makes the obtained PAT LNPs highly suitable for cancer vaccine applications, where robust antigen expression and antigen-specific adaptive immune activation is required.

Compared to BNT162b2 LNPs from Pfizer-BioNTech, our PAT LNP–based vaccines achieved approximately twofold higher antigen mRNA expression within LNs in the mice following subcutaneous administration, which promoted robust antigen cross-presentation. This, in turn, elicited a potent antigen-specific cytotoxic CD8^+^ T cell response, leading to substantial suppression of tumor occurrence and inhibition of pulmonary metastasis in a melanoma model, and a significant delay in tumor recurrence after resection in an orthotopic GBM model. Our strategy based on movable lipids to improve mRNA expression exhibits general applicability and could positively advance local mRNA therapeutics. Future efforts should focus on the development of infrared light–driven movable lipids, which can be achieved by collaborating with chemists to innovate chemical structures. Given the superior tissue penetration depth of infrared light, the movable lipid concept is expected to be extended to deep tissues and organs, including the brains, eyes, joints, and other solid organs. Together, our work transcends current LNP design paradigms by introducing a previously unreported parameter, programmable mechanical movement. This study not only establishes the movable lipid as a structurally innovative class of lipids but also provides a chemical framework for designing next-generation LNP systems with improved delivery efficacy. It is crucial to clarify that the core of our research lies not in LED-driven lipids, but in the foundational design and development of movable lipids. While light serves as a current stimulus, future actuation methods could be diverse. Our long-term vision even includes systems that operate independently of external stimuli, potentially harnessing intrinsic energy gradients such as the proton flux across endosomal membranes, which is a more challenging yet transformative direction.

## MATERIALS AND METHODS

### Materials

2-Aminothiazole, sodium nitrite, sodium hydroxide, phenol, potassium carbonate, and 1-bromononane were purchased from Sigma-Aldrich. 6-((2-Hexyldecanoyl)oxy)-*N*-(6-((2-hexyldecanoyl)oxy)hexyl)-*N*-(4-hydroxybutyl)hexan-1-aminium (ALC-0315), DSPC, 1,2-dioleoyl-*sn*-glycero-3-phosphocholine (DOPC), 1,2-dioleoyl-*sn*-glycero-3-phospho-l-serine (sodium salt) (DOPS), 1,2-dioleoyl-sn-glycero-3-phosphoethanolamine (DOPE), 1,2-dimyristoyl-rac-glycero-3-methoxypolyethylene glycol-2000 (DMG-PEG_2k_), NBD-PE, and Rhod-PE were purchased from Avanti Polar Lipids. d-Luciferin potassium salt was purchased from Thermo Fisher Scientific. DiR and LysoTracker Green were purchased from Invitrogen. Dulbecco’s modified Eagle’s medium growth medium, fetal bovine serum, Dulbecco’s phosphate-buffered saline solution (10 mM, pH 7.2), and trypsin-EDTA (0.25%) were purchased from Gibco. The DC2.4 cell line and B16F10-OVA, EO771, and GL261 tumor cell lines were purchased from the American Type Culture Collection. The antibodies for flow cytometry were purchased from BioLegend and eBioscience.

### Instruments

All ^1^H NMR spectra were recorded on a Bruker AVIII 500 MHz NMR spectrometer (Switzerland) operated in the Fourier transform mode. TEM measurements were performed on a FEI Tecnai Spirit Transmission Electron Microscope. The hydrodynamic sizes of LNPs encapsulating mRNA were characterized in a Nano-ZS Zetasizer (Malvern, USA) using dynamic light scattering. The zeta potentials of LNPs were measured using electrophoretic light scattering. The encapsulation efficiency measurements were conducted by a QuantiFluor assay (Promega, USA). Cell imaging was conducted on a Confocal Laser Scanning Microscope (Zeiss LSM 800, Germany). Flow cytometry analysis was conducted on a NovoCyte Opteon Spectral Flow Cytometer (Novocyte quanteon 4025, USA). In vivo imaging of the mice was conducted on a IVIS imaging system (PerkinElmer, USA). Photoisomerization studies of PAT lipids were performed by a LED light source (Asahi Spectra, CL-1503) equipped with 405- and 525-nm LED heads. In cell and animal experiments, the intensities of blue-violet light and green light are 60 and 40 mW/cm^2^, respectively.

### Synthesis of PAT lipid

2-Aminothiazole (54 mmol) was dissolved in a mixture of distilled water (48 ml) and concentrated hydrochloric acid (16 ml). A solution containing sodium nitrite (58 mmol), phenol (54 mmol), and sodium hydroxide (113 mmol) was added dropwise at 0°C. The resulting mixture was stirred at 4°C for 2 hours in the dark. Upon completion of the reaction, the precipitate was collected by filtration and washed thoroughly with distilled water. The crude product was recrystallized from ethanol to afford intermediate product as a pure solid (7.9 g). Then, acetonitrile (10 ml), intermediate product (5 mmol), 1-bromononane (6 mmol), and potassium carbonate (7.5 mmol) were added sequentially to a round-bottom flask. The reaction mixture was stirred under reflux overnight. After cooling to room temperature, the mixture was filtered to remove solids. The residue was washed with ethyl acetate, and the combined organic layers were concentrated under reduced pressure. The crude product was purified by column chromatography to yield the PAT lipid as a solid (1.2 g).

### The photoisomerization of PAT lipid

PAT lipids were prepared as stock solution (0.5 mg/ml) in anhydrous acetonitrile. The samples (0.05 mg/ml) were irradiated with violet-blue (405 nm) and green light (525 nm), respectively.

### Preparation of different LNPs

The LNPs were formulated with ALC-0315 lipid, PAT lipid, cholesterol, DSPC, and DMG-PEG_2k_ using an ethanol dilution method. Adding PAT lipids as the fifth component does not affect the other original components. The formulations with different percentages of PAT lipids (from 0 to 50% molar ratio) were prepared below. BNT LNP: The molar ratio of ALC-0315 lipid/cholesterol/DSPC/DMG-PEG_2k_ was 50/38.5/10/1.5; PAT(10%) LNP: The molar ratio of ALC-0315 lipid/PAT lipid/cholesterol/DSPC/DMG-PEG_2k_ was 37.5/10/12.5/38.5/10/1.5; PAT(20%) LNP: The molar ratio of ALC-0315 lipid/PAT lipid/cholesterol/DSPC/DMG-PEG_2k_ was 37.5/20/12.5/38.5/10/1.5; PAT(30%) LNP: The molar ratio of ALC-0315 lipid/PAT lipid/cholesterol/DSPC/DMG-PEG_2k_ was 37.5/30/12.5/38.5/10/1.5; PAT(40%) LNP: The molar ratio of ALC-0315 lipid/PAT lipid/cholesterol/DSPC/DMG-PEG_2k_ was 37.5/40/12.5/38.5/10/1.5; PAT(50%) LNP: The molar ratio of ALC-0315 lipid/PAT lipid/cholesterol/DSPC/DMG-PEG_2k_ was 37.5/50/12.5/38.5/10/1.5.

### Uptake of mRNA-LNPs and mGFP expression in DC2.4 cells

Flow cytometry was used to analyze the cellular uptake of Cy5-labeled mRNA-loaded LNPs and their ability to facilitate mGFP expression in the DC2.4 cell line. For cellular uptake evaluation, DC 2.4 cells (5 × 10^4^ cells per well) were seeded in a 12-well plate and incubated overnight for cell attachment. Different inhibitors, including nystatin, chlorpromazine, and amiloride, were used to investigate the internalization pathways. Cy5-labeled mRNA-loaded LNPs (mRNA, 1 μg/ml) were incubated with DC 2.4 cells for 3 hours in the presence of different inhibitors. The cells were washed three times with PBS and then digested with 0.25% trypsin for flow cytometry analysis. For mGFP expression evaluation, Cy5-labeled mRNA-loaded LNPs were incubated with DC2.4 cells for 3 hours. The cells were then irradiated with LED light for the first 10 min of every hour (i.e., 60 to 70, 120 to 130, and 180 to 190 min), using 10-s alternating cycles of 405- and 525-nm wavelengths. After replacing with fresh culture medium, the cells were then incubated for an additional 21 hours, washed three times with PBS, and subsequently digested with 0.25% trypsin for flow cytometry analysis. All data were analyzed using Flowjo V10.

### Endosomal escape measurements

SLEEQ assay is based on a highly sensitive bioluminescent split luciferase system that is composed of two subunits: a large BiT protein (LgBiT, 17.8 kDa) and a high-affinity complementary peptide (HiBiT, 1.3 kDa). When separated, these fragments have no luminescent activity, but when brought together, they form a functional enzyme that binds to a substrate that produces bright luminescence. We first transfected the cells with an LgBiT expression vector. LgBiT-expressing cells were then incubated with HiBiT-loaded LNPs and received LED irradiation. The release of HiBiT into the cytoplasm generated a luminescent signal, where the signal intensity correlated with the efficiency of endosomal escape.

### Hemolysis assay

Mouse RBCs were isolated from freshly collected whole blood by centrifugation at 10,000*g* for 5 min, followed by washing five times with PBS. A hemolysis assay was conducted by coincubating RBC and LNPs at pH 5.5 to evaluate the ability to disrupt the endosomal membranes.

### FRET assay

FRET assay was conducted to evaluate lipid fusion and/or disruption. Endosomal mimicking liposomes were prepared via mixing DOPS, DOPC, DOPE, NBD-PE, and Rhod-PE in a molar ratio of 25/25/48/1/1 in chloroform. The mixture was subjected to rotary evaporation and vacuum drying to obtain a thin lipid film. The dried film was then hydrated in PBS and sonicated for 10 min, with the total lipid concentration fixed at 1 mM. Two FRET probes were incorporated into one liposome, resulting in attenuated NBD fluorescence due to FRET to Rhod. Upon fusion and/or disruption, the NBD fluorescence increased due to the enhanced distance between the probes. Fluorescence intensities were recorded at E_x_/E_m_ = 465/520 nm using a microplate reader, and the FRET efficiency was calculated.

### mLuc expression in the inguinal LNs

All animal protocols were approved by the Laboratory Animal Welfare and Ethics Committee of Zhejiang University, the Institute of Radiation Medicine, Chinese Academy of Medical Sciences, and Harbin Medical University (ZJU20250976; IRM/2-IACUC-2403-063; GZR2023-02). Male C57BL/6 mice (the Jackson Laboratory) at 6 to 8 weeks old were housed in a specific pathogen–free–grade animal facility with air humidity 40 to 70%, ambient temperature (22° ± 2°C), and a 12-hour dark/12-hour light cycle. Following the subcutaneous injection of different mLuc-loaded DiR-labeled LNPs, the inguinal LNs of the mice were exposed to LED irradiation during the first 15 min of each of the second, third, and fourth hours (i.e., 60 to 75, 120 to 135, and 180 to 195 min). The light source alternated between two wavelengths (i.e., 405 and 525 nm) every 15 s. After 5 hours, 100 μl of d-Luciferin potassium salt solution (15 mg/ml) was intraperitoneally administered. After 10 min, the biodistribution of DiR-labeled LNPs and mLuc expression were visualized using an IVIS imaging system.

### mCre expression the inguinal LNs in Ai14 reporter mice

mCre-loaded LNPs (mCre, 5 μg per mouse) were subcutaneously injected into the Ai14 mice (the Jackson Laboratory, 6 to 8 weeks old). The inguinal LNs of the mice were exposed to LED irradiation during the first 15 min of each of the second, third, and fourth hours (i.e., 60 to 75, 120 to 135, and 180 to 195 min). After 72 hours of injection, the mice were euthanized, and the LNs were collected. Single-cell suspensions were prepared by grinding and filtrating through a 70-μm strainer. A total of 1 × 10^6^ cells were washed with PBS and stained with live/dead viability dye, followed by washing with flow cytometry staining buffer and incubating with FcR blocking reagent. Then, the cells were stained with 100 μl of flow cytometry staining buffer containing different fluorophore-conjugated antibodies of interest at the recommended concentration for 1 hour at 4°C. The cells were washed twice with staining buffer for flow cytometry analysis. The gating strategy is shown in fig. S11.

### Antigen-specific cross-presentation assay

BMDCs were generated from the bone marrow of male C57BL/6 mice. Flow cytometry analysis was performed to study the antigen cross-presentation in BMDCs. Different mOVA-loaded LNPs (mOVA, 1 μg/ml) were incubated with BMDCs for 3 hours. The cells were then irradiated with LED light for the first 10 min of every hour (i.e., 60 to 70, 120 to 130, and 180 to 190 min), using 10-s alternating cycles of 405- and 525-nm wavelengths. After replacing with fresh culture medium, the cells were then incubated for an additional 21 hours. BMDCs were then collected and stained with the flow cytometry staining buffer containing different fluorophore-conjugated antibody of interest at the recommended concentration for 1 hour at 4°C.

### Antigen-specific T cell–dependent immune responses

Male mice at 6 to 8 weeks old were subcutaneously vaccinated with mOVA-loaded LNPs (mOVA, 15 μg per mouse) and received LED irradiation. Briefly, mice received a prime vaccination on day 0 with booster doses on days 5 and 10. The inguinal LNs of the mice were exposed to LED irradiation during the first 15 min of each of the second, third, and fourth hours (i.e., 60 to 75, 120 to 135, and 180 to 195 min) after each vaccination. LNs were collected on day 12 to assess antigen cross-presentation and OVA-specific CD8^+^ T cell activation. Single-cell suspensions were prepared, and seeded in a 12-well plate (5 × 10^4^ cells per well), followed by stimulating with OVA257 peptides at 2 μg/ml for 6 hours. After stimulation, 1 × 10^6^ cells were washed with PBS. The cells were washed by flow cytometry staining buffer and incubated with FcR blocking reagent. Then, the cells were stained with 100 μl of flow cytometry staining buffer containing different fluorophore-conjugated antibodies of interest at the recommended concentration for 1 hour at 4°C.

### Prevention of the melanoma growth in a mouse model

Male C57BL/6 mice at 6 to 8 weeks old and B16F10-OVA tumor cells were used. The mice were randomly divided into five groups (*n* = 5). After vaccination and LED irradiation, the mice were challenged by subcutaneous injection of B16F10-OVA tumor cells (1 × 10^6^ per mouse) into the left flank on day 15. The mice were euthanized when exhibiting signs of impaired health (>15% loss of body weight) or when the volume of the tumor exceeded 1.5 cm^3^.

### Prevention of cognate tumor growth

The male C57BL/6 mice at 6 to 8 weeks old were randomly divided into two groups (*n* = 3). The mice treated with PBS was used as control. After vaccination and LED irradiation, mice were challenged with B16F10-OVA and noncognate E0771 murine mammary tumor cells via subcutaneous injection on day 15. Briefly, B16F10-OVA tumor cells (1 × 10^6^ per mouse) were inoculated into the left flank, while E0771 tumor cells (1 × 10^6^ per mouse) were inoculated into the opposite flank. The growth of tumors was monitored.

### Prevention of tumor metastasis to lungs

Male C57BL/6 mice at 6 to 8 weeks old were randomly divided into five groups (*n* = 3). After vaccination and LED irradiation, mice were intravenously challenged with B16F10-OVA tumor cells (1 × 10^6^ per mouse) via the tail vein on day 15. On day 35, the mice were euthanized and the lung tissues were collected for histological analysis.

### Prevention of postoperative recurrence of GBM

Male C57BL/6 mice at 6 to 8 weeks old were used to construct an orthotopic GBM-bearing model via stereotactically injection using GL261-OVA-Luc tumor cells (5 × 10^5^ per mouse). The GBM growth was monitored by the IVIS imaging system. Then, the mice were divided into four groups (*n* = 5). Fourteen days after inoculation, mice carrying GL261-OVA-Luc cells were operated on a warmed heating pad (37°C). According to the change in bioluminescence intensity, whether the tumor is effectively removed can be judged. The mice were subcutaneously vaccinated with mOVA-loaded LNPs (mOVA, 15 μg per mouse) in the interscapular region and received LED irradiation. The axillary LNs of the mice were exposed to LED irradiation during the first 15 min of each of the second, third, and fourth hours (i.e., 60 to 75, 120 to 135, and 180 to 195 min) after each vaccination. aPD-1 (200 μg per dose; three doses) were administered by intraperitoneal injection. The development of intracranial tumors was measured by the IVIS imaging system (every 5 days). Mice bearing GBM were considered to be under moribund conditions when significant weight loss (>15% loss of body weight) and neurological symptoms such as ataxia and seizures were observed. Mice will be euthanized by injection of pentobarbital (200 mg/kg) when they suffer from the above symptoms. Flow analysis was performed according to the protocol described earlier. The levels of proinflammatory cytokines and proinflammatory chemokines in the blood were measured with enzyme-linked immunosorbent assay kits according to the manufacturer’s instructions.

### Antibody list

The following antibodies were used in the study: eFluor 450–anti-mouse CD19 antibody (eBioscience, catalog no. 48-0193-82, clone no. 1D3), FITC–anti-mouse CD3 antibody (BioLegend, catalog no. 100203-1, clone no. 17A2), APC–anti-mouse NK1.1 antibody (BioLegend, catalog no. 156506, clone no. S17016D), PE-Cy7–anti-mouse CD11c antibody (BioLegend, catalog no. 117318, clone no. N418), Super Bright 600–anti-mouse CD11b antibody (eBioscience, catalog no. 63–0112-82, clone no. M1/70), PerCP-Cy5.5–anti-mouse Ly6G antibody (BioLegend, catalog no. 127616, clone no. 1A8), PE-Cy7–anti-mouse CD8a antibody (BioLegend, catalog no. 100722, clone no. 53-6.7), APC–anti-mouse-H2kb-SIINFEKL antibody (eBioscience, catalog no. 17-5743-82, clone no. eBio25-D1.16), BV421-H2Kb OVA tetramer-SIINFEKL (COSMO Bio USA, catalog no. MBL-TS-5001-4C), Super Bright 600–anti-mouse CD62L antibody (eBioscience, catalog no. 63-0621-80, clone no. MEL-14), AF700–anti-mouse CD44 antibody (BioLegend, catalog no. 103026, clone no. IM7), FITC–anti-mouse CD45 antibody (BioLegend, catalog no. 103108, clone no. 30-F11), and PerCP-Cy5.5-anti-mouse CD3 antibody (BioLegend, catalog no. 100218, clone no. 17A2).

### Statistical analyses

Statistical analyses were conducted with GraphPad Prism (8.0) software. Data are presented as mean ± SD. The statistical analysis methods and experiments performed in biological replicates are described in each figure caption. No statistical method was used to predetermine sample size. No data were excluded from the analyses.

## References

[1] M. Verma, I. Ozer, W. Xie, R. Gallagher, A. Teixeira, M. Choy, The landscape for lipid-nanoparticle-based genomic medicines. Nat. Rev. Drug Discov. 22, 349–350 (2023). 36627441 10.1038/d41573-023-00002-2

[2] Y. Zong, Y. Lin, T. Wei, Q. Cheng, Lipid nanoparticle (LNP) enables mRNA delivery for cancer therapy. Adv. Mater. 35, 2303261 (2023). 10.1002/adma.202303261 37196221

[3] X. Han, M.-G. Alameh, K. Butowska, J. J. Knox, K. Lundgreen, M. Ghattas, N. Gong, L. Xue, Y. Xu, M. Lavertu, Adjuvant lipidoid-substituted lipid nanoparticles augment the immunogenicity of SARS-CoV-2 mRNA vaccines. Nat. Nanotechnol. 18, 1105–1114 (2023). 37365276 10.1038/s41565-023-01404-4

[4] X. Huang, N. Kong, X. Zhang, Y. Cao, R. Langer, W. Tao, The landscape of mRNA nanomedicine. Nat. Med. 28, 2273–2287 (2022). 36357682 10.1038/s41591-022-02061-1

[5] S. Chen, X. Huang, Y. Xue, E. Álvarez-Benedicto, Y. Shi, W. Chen, S. Koo, D. J. Siegwart, Y. Dong, W. Tao, Nanotechnology-based mRNA vaccines. Nat. Rev. Methods Primers 3, 63 (2023). 40747084 10.1038/s43586-023-00246-7 PMC12312698

[6] C. Liu, Q. Shi, X. Huang, S. Koo, N. Kong, W. Tao, mRNA-based cancer therapeutics. Nat. Rev. Cancer 23, 526–543 (2023). 37311817 10.1038/s41568-023-00586-2

[7] F. P. Polack, S. J. Thomas, N. Kitchin, J. Absalon, A. Gurtman, S. Lockhart, J. L. Perez, G. Pérez Marc, E. D. Moreira, C. Zerbini, Safety and efficacy of the BNT162b2 mRNA Covid-19 vaccine. N. Engl. J. Med. 383, 2603–2615 (2020). 33301246 10.1056/NEJMoa2034577 PMC7745181

[8] L. R. Baden, H. M. El Sahly, B. Essink, K. Kotloff, S. Frey, R. Novak, D. Diemert, S. A. Spector, N. Rouphael, C. B. Creech, Efficacy and safety of the mRNA-1273 SARS-CoV-2 vaccine. N. Engl. J. Med. 384, 403–416 (2021). 33378609 10.1056/NEJMoa2035389 PMC7787219

[9] X. Hou, T. Zaks, R. Langer, Y. Dong, Lipid nanoparticles for mRNA delivery. Nat. Rev. Mater. 6, 1078–1094 (2021). 34394960 10.1038/s41578-021-00358-0 PMC8353930

[10] D. Song, Y. Zhao, Z. Wang, Q. Xu, Tuning lipid nanoparticles for RNA delivery to extrahepatic organs. Adv. Mater. 36, e2401445 (2024). 39233550 10.1002/adma.202401445 PMC11530311

[11] Z. Lian, L. Zheng, S. Liu, J. Zhang, J. Zhou, J. Wu, S. Ouyang, J. Li, H. Yang, Breaking endosomal barriers: Thiol-mediated uptake lipid nanoparticles for efficient mRNA vaccine delivery. J. Am. Chem. Soc. 147, 31530–31540 (2025). 40694665 10.1021/jacs.5c05367

[12] X. Han, H. Zhang, K. Butowska, K. L. Swingle, M.-G. Alameh, D. Weissman, M. J. Mitchell, An ionizable lipid toolbox for RNA delivery. Nat. Commun. 12, 7233 (2021). 34903741 10.1038/s41467-021-27493-0 PMC8668901

[13] S. Liu, Q. Cheng, T. Wei, X. Yu, L. T. Johnson, L. Farbiak, D. J. Siegwart, Membrane-destabilizing ionizable phospholipids for organ-selective mRNA delivery and CRISPR–Cas gene editing. Nat. Mater. 20, 701–710 (2021). 33542471 10.1038/s41563-020-00886-0 PMC8188687

[14] Y. Zhao, D. Song, Z. Wang, Q. Huang, F. Huang, Z. Ye, D. Wich, M. Chen, J. Khirallah, S. Gao, Antitumour vaccination via the targeted proteolysis of antigens isolated from tumour lysates. Nat. Biomed. Eng. 9, 234–248 (2025). 39609559 10.1038/s41551-024-01285-5

[15] N. Gong, Y. Zhang, X. Teng, Y. Wang, S. Huo, G. Qing, Q. Ni, X. Li, J. Wang, X. Ye, Proton-driven transformable nanovaccine for cancer immunotherapy. Nat. Nanotechnol. 15, 1053–1064 (2020). 33106640 10.1038/s41565-020-00782-3 PMC7719078

[16] J. Gilleron, W. Querbes, A. Zeigerer, A. Borodovsky, G. Marsico, U. Schubert, K. Manygoats, S. Seifert, C. Andree, M. Stöter, Image-based analysis of lipid nanoparticle–mediated siRNA delivery, intracellular trafficking and endosomal escape. Nat. Biotechnol. 31, 638–646 (2013). 23792630 10.1038/nbt.2612

[17] Y. Eygeris, M. Gupta, J. Kim, G. Sahay, Chemistry of lipid nanoparticles for RNA delivery. Acc. Chem. Res. 55, 2–12 (2021). 34850635 10.1021/acs.accounts.1c00544

[18] Y. Zhao, Z. Tian, J. Wang, M. Cui, Z. Cao, P. Liu, R. Li, S. Cai, Y. Hu, Y. Ma, Replacing cholesterol and PEGylated lipids with zwitterionic ionizable lipids in LNPs for spleen-specific mRNA translation. Sci. Adv. 11, eady6460 (2025). 41061074 10.1126/sciadv.ady6460 PMC12506999

[19] S. Zhao, K. Gao, H. Han, M. Stenzel, B. Yin, H. Song, A. Lawanprasert, J. E. Nielsen, R. Sharma, O. H. Arogundade, Acid-degradable lipid nanoparticles enhance the delivery of mRNA. Nat. Nanotechnol. 19, 1702–1711 (2024). 39179796 10.1038/s41565-024-01765-4 PMC12479011

[20] S. Luozhong, P. Liu, R. Li, Z. Yuan, E. Debley, Y. Chen, Y. Hu, Z. Cao, M. Cui, K. McIlhenny, Poly (carboxybetaine) lipids enhance mRNA therapeutics efficacy and reduce their immunogenicity. Nat. Mater. 24, 1852–1861 (2025). 40442447 10.1038/s41563-025-02240-8 PMC12354250

[21] Y. Zhao, Z. Qu, M. Paloncýová, Z. Wang, B. Weng, Y. Zhao, L. Liu, D. Song, D. Wich, M. Otyepka, Spatial conformation of ionizable lipids regulates endosomal membrane disruption. J. Am. Chem. Soc. 147, 38265–38274 (2025). 41069017 10.1021/jacs.5c10908

[22] L. Xue, G. Zhao, N. Gong, X. Han, S. J. Shepherd, X. Xiong, Z. Xiao, R. Palanki, J. Xu, K. L. Swingle, Combinatorial design of siloxane-incorporated lipid nanoparticles augments intracellular processing for tissue-specific mRNA therapeutic delivery. Nat. Nanotechnol. 20, 132–143 (2025). 39354147 10.1038/s41565-024-01747-6 PMC12207990

[23] G. Saper, H. Hess, Synthetic systems powered by biological molecular motors. Chem. Rev. 120, 288–309 (2020). 31509383 10.1021/acs.chemrev.9b00249

[24] M. Schliwa, G. Woehlke, Molecular motors. Nature 422, 759–765 (2003). 12700770 10.1038/nature01601

[25] Z. Li, J. Wang, H. Zhuo, Q. Li, Q. Huang, C. Tang, W. Zhai, Y. Liu, Y. Zhao, Visible light-driven membrane-bound compartment for precise regulation of enzyme activity. Angew. Chem. Int. Ed. Engl. 64, e202513676 (2025). 40926401 10.1002/anie.202513676

[26] F. Lancia, A. Ryabchun, N. Katsonis, Life-like motion driven by artificial molecular machines. Nat. Rev. Chem. 3, 536–551 (2019).

[27] T. Dang, Z.-Y. Zhang, T. Li, Visible-light-activated heteroaryl azoswitches: Toward a more colorful future. J. Am. Chem. Soc. 146, 19609–19620 (2024). 38991225 10.1021/jacs.4c03135

[28] S. Lee, H. J. Kim, J.-H. Choi, H. J. Jang, H. B. Cho, H. R. Kim, J. I. Park, K. S. Park, K.-H. Park, Light emitting diode (LED) irradiation of liposomes enhances drug encapsulation and delivery for improved cancer eradication. J. Control. Release 368, 756–767 (2024). 38499090 10.1016/j.jconrel.2024.03.027

[29] K. A. Ryu, C. M. Kaszuba, N. B. Bissonnette, R. C. Oslund, O. O. Fadeyi, Interrogating biological systems using visible-light-powered catalysis. Nat. Rev. Chem. 5, 322–337 (2021). 37117838 10.1038/s41570-021-00265-6

[30] J. Xu, X. Zhou, Z. Gao, Y. Y. Song, P. Schmuki, Visible-light-triggered drug release from TiO_2_ nanotube arrays: A controllable antibacterial platform. Angew. Chem. Int. Ed. Engl. 55, 593–597 (2016). 26592984 10.1002/anie.201508710

[31] M. H. Qi, D. D. Wang, W. Qian, Z. L. Zhang, Y. W. Ao, J. M. Li, S. W. Huang, High-efficiency gold nanoaggregates for NIR LED-driven sustained mild photothermal therapy achieving complete tumor eradication and immune enhancement. Adv. Mater. 37, e2412191 (2025). 39676384 10.1002/adma.202412191

[32] M. Oungeun, S. Wanichwecharungruang, E. Miyako, Wireless light-emitting diode-driven functional microneedle devices for skin cancer therapy. Adv. Ther. 7, 2400233 (2024).

[33] C. Ash, M. Dubec, K. Donne, T. Bashford, Effect of wavelength and beam width on penetration in light-tissue interaction using computational methods. Lasers Med. Sci. 32, 1909–1918 (2017). 28900751 10.1007/s10103-017-2317-4 PMC5653719

[34] J. C. Wei, G. A. Edwards, D. J. Martin, H. Huang, M. L. Crichton, M. A. Kendall, Allometric scaling of skin thickness, elasticity, viscoelasticity to mass for micro-medical device translation: From mice, rats, rabbits, pigs to humans. Sci. Rep. 7, 15885 (2017). 29162871 10.1038/s41598-017-15830-7 PMC5698453

[35] P. Delgado-López, E. Corrales-García, Survival in glioblastoma: A review on the impact of treatment modalities. Clin. Transl. Oncol. 18, 1062–1071 (2016). 26960561 10.1007/s12094-016-1497-x

[36] R. S. Angom, N. M. R. Nakka, S. Bhattacharya, Advances in glioblastoma therapy: An update on current approaches. Brain Sci. 13, 1536 (2023). 38002496 10.3390/brainsci13111536 PMC10669378

[37] H. Liu, K. D. Moynihan, Y. Zheng, G. L. Szeto, A. V. Li, B. Huang, D. S. Van Egeren, C. Park, D. J. Irvine, Structure-based programming of lymph-node targeting in molecular vaccines. Nature 507, 519–522 (2014). 24531764 10.1038/nature12978 PMC4069155

[38] M. Tozuka, T. Oka, N. Jounai, G. Egawa, K. J. Ishii, K. Kabashima, F. Takeshita, Efficient antigen delivery to the draining lymph nodes is a key component in the immunogenic pathway of the intradermal vaccine. J. Dermatol. Sci. 82, 38–45 (2016). 26674124 10.1016/j.jdermsci.2015.11.008

[39] M. Buckley, M. Araínga, L. Maiorino, I. S. Pires, B. Kim, K. K. Michaels, J. Dye, K. Qureshi, Y. J. Zhang, H. Mak, Visualizing lipid nanoparticle trafficking for mRNA vaccine delivery in non-human primates. Mol. Ther. 33, 1105–1117 (2025). 39797396 10.1016/j.ymthe.2025.01.008 PMC11897755

[40] J. Rejman, A. Bragonzi, M. Conese, Role of clathrin-and caveolae-mediated endocytosis in gene transfer mediated by lipo-and polyplexes. Mol. Ther. 12, 468–474 (2005). 15963763 10.1016/j.ymthe.2005.03.038

[41] I. R. Nabi, P. U. Le, Caveolae/raft-dependent endocytosis. J. Cell Biol. 161, 673–677 (2003). 12771123 10.1083/jcb.200302028 PMC2199359

[42] J. P. Lim, P. A. Gleeson, Macropinocytosis: An endocytic pathway for internalising large gulps. Immunol. Cell Biol. 89, 836–843 (2011). 21423264 10.1038/icb.2011.20

[43] J. Adler, I. Parmryd, Quantifying colocalization by correlation: The Pearson correlation coefficient is superior to the Mander’s overlap coefficient. Cytometry A 77, 733–742 (2010). 20653013 10.1002/cyto.a.20896

[44] T. Lu, F. Chen, Multiwfn: A multifunctional wavefunction analyzer. J. Comput. Chem. 33, 580–592 (2012). 22162017 10.1002/jcc.22885

[45] Z. Liu, J. Wu, N. Wang, Y. Lin, R. Song, M. Zhang, B. Li, Structure-guided design of endosomolytic chloroquine-like lipid nanoparticles for mRNA delivery and genome editing. Nat. Commun. 16, 4241 (2025). 40335474 10.1038/s41467-025-59501-y PMC12058976

[46] G. Mahalingam, H. K. Rachamalla, P. Arjunan, K. V. Karuppusamy, Y. Periyasami, A. Mohan, K. Subramaniyam, V. Rajendran, M. Moorthy, G. M. Varghese, SMART-lipid nanoparticles enabled mRNA vaccine elicits cross-reactive humoral responses against the omicron sub-variants. Mol. Ther. 32, 1284–1297 (2024). 38414245 10.1016/j.ymthe.2024.02.028 PMC11081802

[47] M. A. Beach, S. L. Teo, M. Z. Chen, S. A. Smith, C. W. Pouton, A. P. Johnston, G. K. Such, Quantifying the endosomal escape of pH-responsive nanoparticles using the split luciferase endosomal escape quantification assay. ACS Appl. Mater. Interfaces 14, 3653–3661 (2021). 34964593 10.1021/acsami.1c18359

[48] S. L. Teo, J. J. Rennick, D. Yuen, H. Al-Wassiti, A. P. Johnston, C. W. Pouton, Unravelling cytosolic delivery of cell penetrating peptides with a quantitative endosomal escape assay. Nat. Commun. 12, 3721 (2021). 34140497 10.1038/s41467-021-23997-x PMC8211857

[49] Y. Fang, B. Ma, Z. Zhao, Z. Xuan, Y. Wang, C. Huang, M. Zhang, L. Zhou, F. Yao, K. Li, mRNA-based FRET-FLIM imaging platform for quantifying lipid nanoparticle endosomal escape and membrane damage. J. Am. Chem. Soc. 147, 30907–30923 (2025). 40814995 10.1021/jacs.5c07897

[50] A. Schroeder, M. S. Goldberg, C. Kastrup, Y. Wang, S. Jiang, B. J. Joseph, C. G. Levins, S. T. Kannan, R. Langer, D. G. Anderson, Remotely activated protein-producing nanoparticles. Nano Lett. 12, 2685–2689 (2012). 22432731 10.1021/nl2036047 PMC3388722

[51] J. Chen, Z. Ye, C. Huang, M. Qiu, D. Song, Y. Li, Q. Xu, Lipid nanoparticle-mediated lymph node–targeting delivery of mRNA cancer vaccine elicits robust CD8+ T cell response. Proc. Natl. Acad. Sci. U.S.A. 119, e2207841119 (2022). 35969778 10.1073/pnas.2207841119 PMC9407666

[52] S. Singh, D. Dey, D. Barik, I. Mohapatra, S. Kim, M. Sharma, S. Prasad, P. Wang, A. Singh, G. Singh, Glioblastoma at the crossroads: Current understanding and future therapeutic horizons. Signal Transduct. Target. Ther. 10, 213 (2025). 40628732 10.1038/s41392-025-02299-4 PMC12238593

[53] K. Feng, X. Zhang, J. Li, M. Han, J. Wang, F. Chen, Z. Yi, L. Di, R. Wang, Neoantigens combined with in situ cancer vaccination induce personalized immunity and reshape the tumor microenvironment. Nat. Commun. 16, 5074 (2025). 40450037 10.1038/s41467-025-60448-3 PMC12126593

