# Supplementary material for: Mechanical movements generated by movable lipids break endosomal barriers for enhanced mRNA therapeutics
Source: Sci Adv. 2026 Jul 1;12(27):eaef1695. doi: 10.1126/sciadv.aef1695 (PMC13322231; doi:10.1126/sciadv.aef1695)
Supplement: Supplementary file 1 — Figs. S1 to S11 [file sciadv.aef1695_sm.pdf]

Science Advances
AAAS

Supplementary Materials for

Mechanical movements generated by movable lipids break endosomal barriers for enhanced mRNA therapeutics

Zilu Li *et al.*

Corresponding author: Yumiao Chen, yumiaoc7@163.com; Jinquan Cai, caijinquan666777@126.com; Fan Huang, huangfan@irm-cams.ac.cn; Jianqing Gao, gaojianqing@zju.edu.cn; Yu Zhao, yzhaopharm@zju.edu.cn

Sci. Adv. **12**, eaef1695 (2026)
DOI: 10.1126/sciadv.aef1695

This PDF file includes:

Figs. S1 to S11

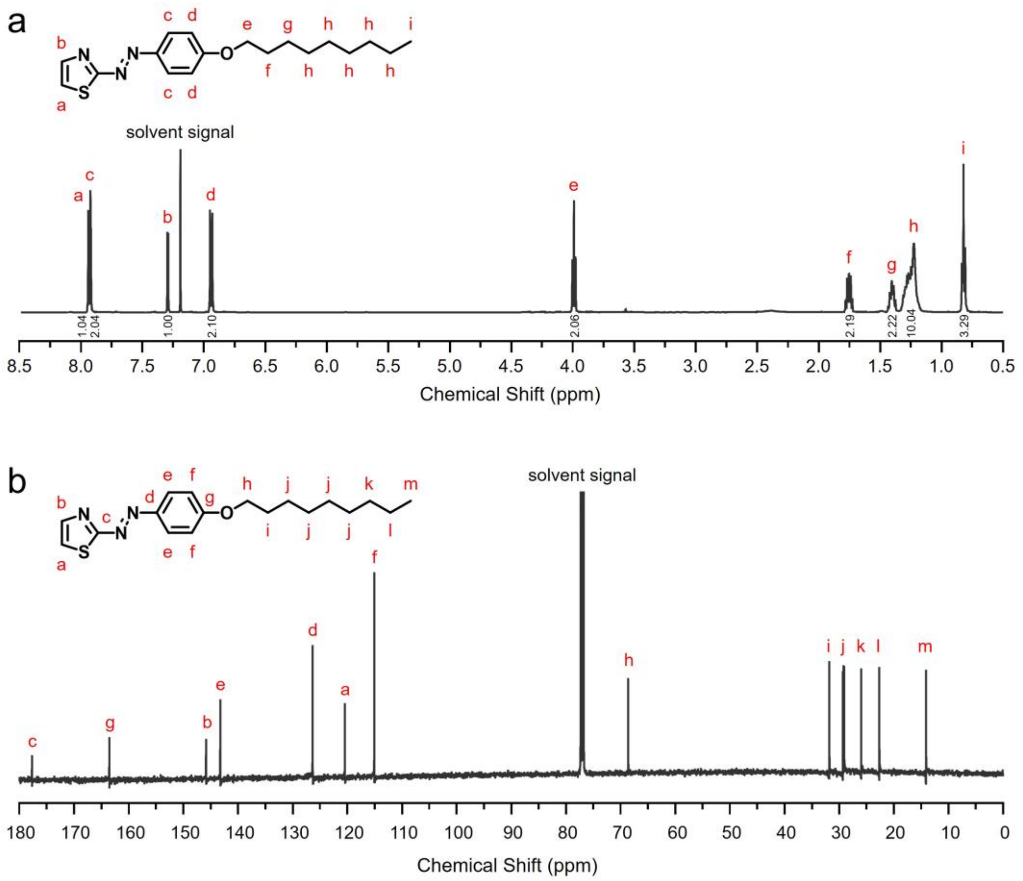

Fig. S1. Characterization of phenylazothiazole (PAT) lipid. a) ^{1}H NMR spectrum of PAT lipid in $CDCl_3$, 500 MHz. b) ^{13}C NMR spectrum of PAT lipid in $CDCl_3$.

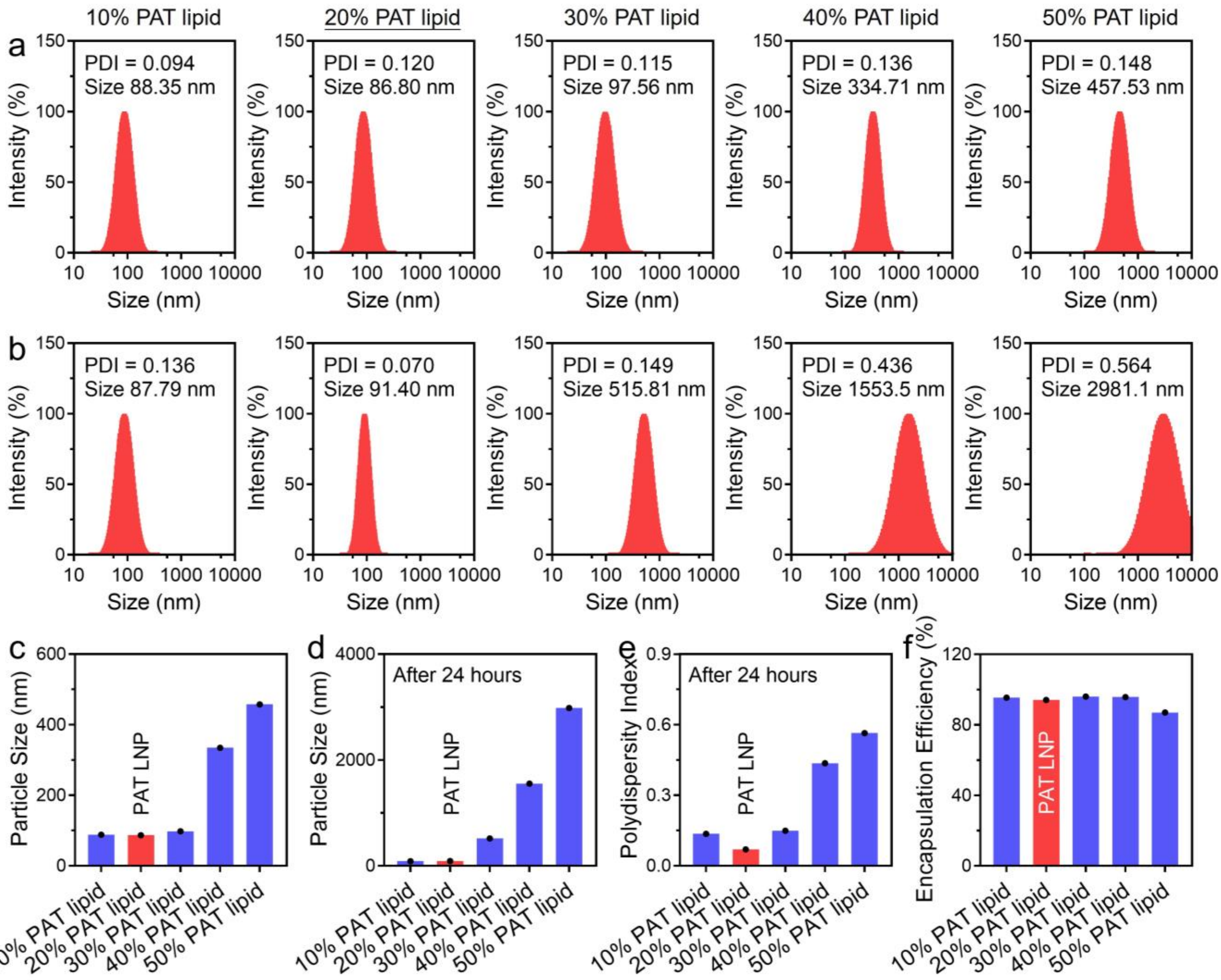

Fig. S2. Characterization of PAT LNP. a) and c) Hydrodynamic diameters of the LNPs containing different percentages of PAT lipids. b) and d) Monitoring of size changes in different LNPs after 24-hour incubation at 37 °C. e) Polydispersity index (PDI) values of different LNPs after 24-hour incubation at 37 °C. f) Encapsulation efficiencies of the different LNPs.

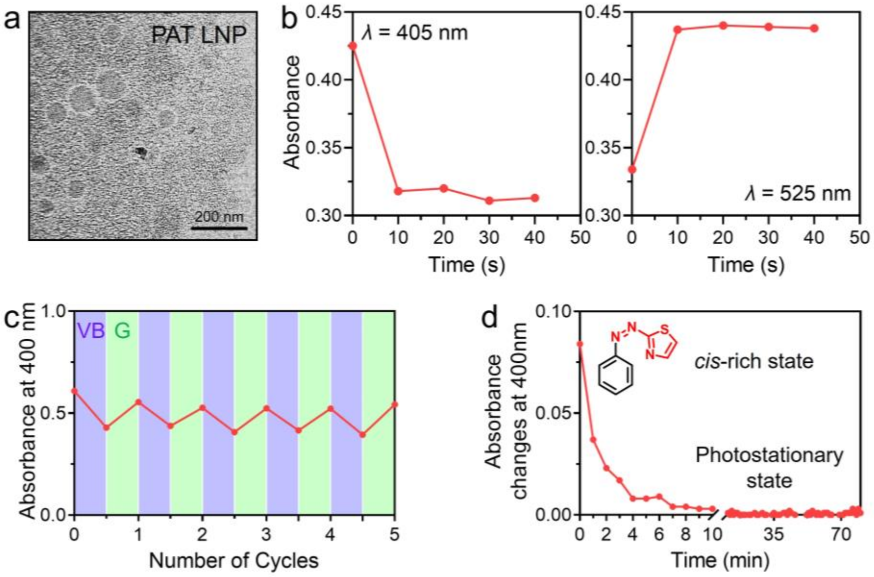

Fig. S3. Reversible photoisomerization of PAT lipid. a) Representative transmission electron microscopy (TEM) image of PAT LNP. Scale bar, 200 nm. b) UV−Vis absorption spectrum of PAT lipids in PAT LNPs before and after irradiation with violet-blue light (405 nm for 5 seconds) and green light (525 nm for 5 seconds). c) Repeated irradiation with alternating wavelengths led to reversible changes in absorbance at 400 nm. d) Thermal relaxation of cis-isomers of PAT lipids in PAT LNPs.

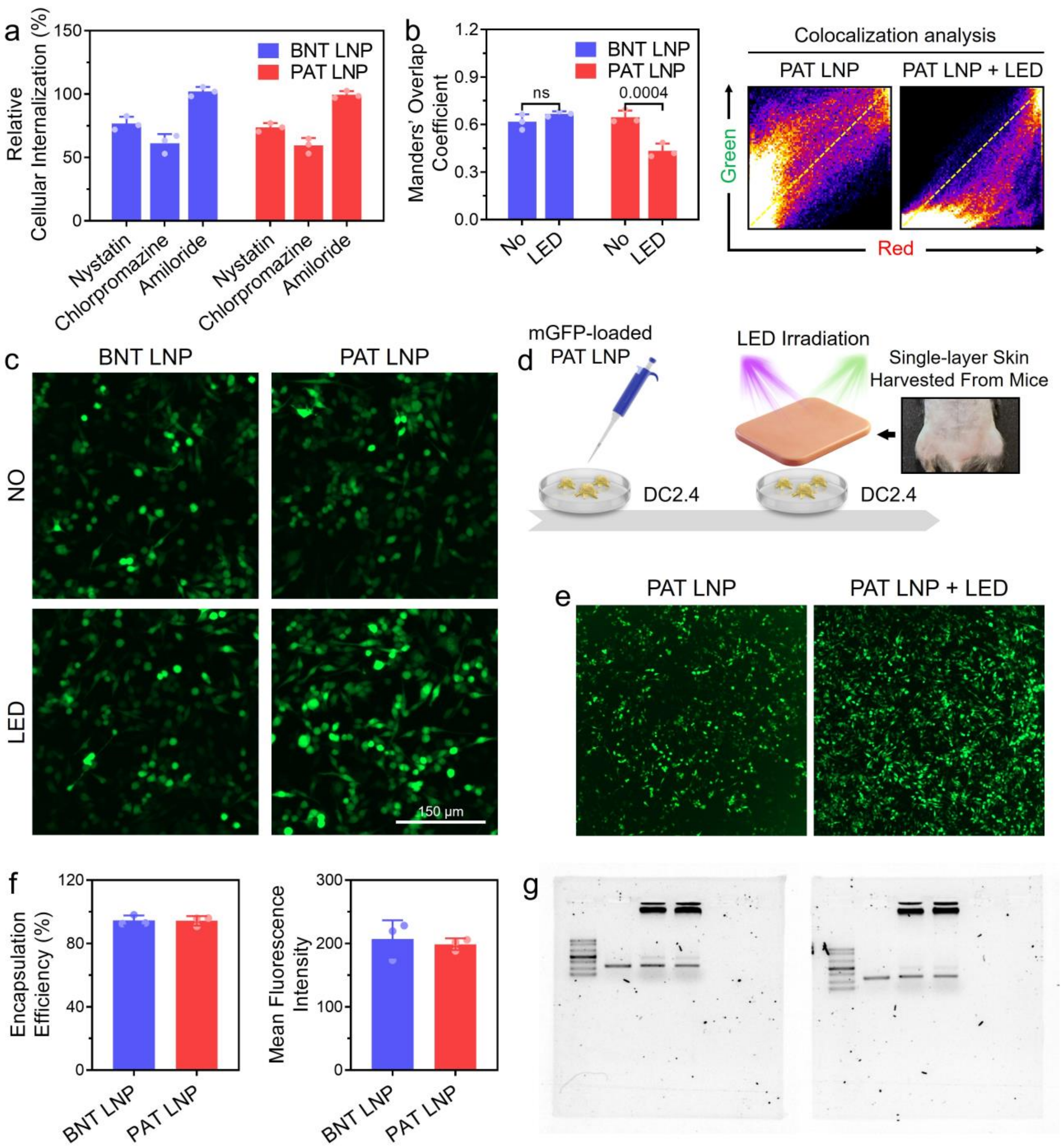

Fig. S4. Mechanical movements generated by PAT lipids facilitate mGFP expression. a) The internalization of different LNPs in DC2.4 cells in the presence of different inhibitors. b) Manders' Overlap Coefficient values. c) GFP expression in DC2.4 cells after different treatments. d) Schematic illustration of the experimental design. e) GFP expression in DC2.4 cells after different treatments. f) Encapsulation efficiency of different LNPs; Cellular uptake of different LNPs by DC2.4 cells. g) Uncropped agarose gel electrophoresis images showed in Fig. 2I.

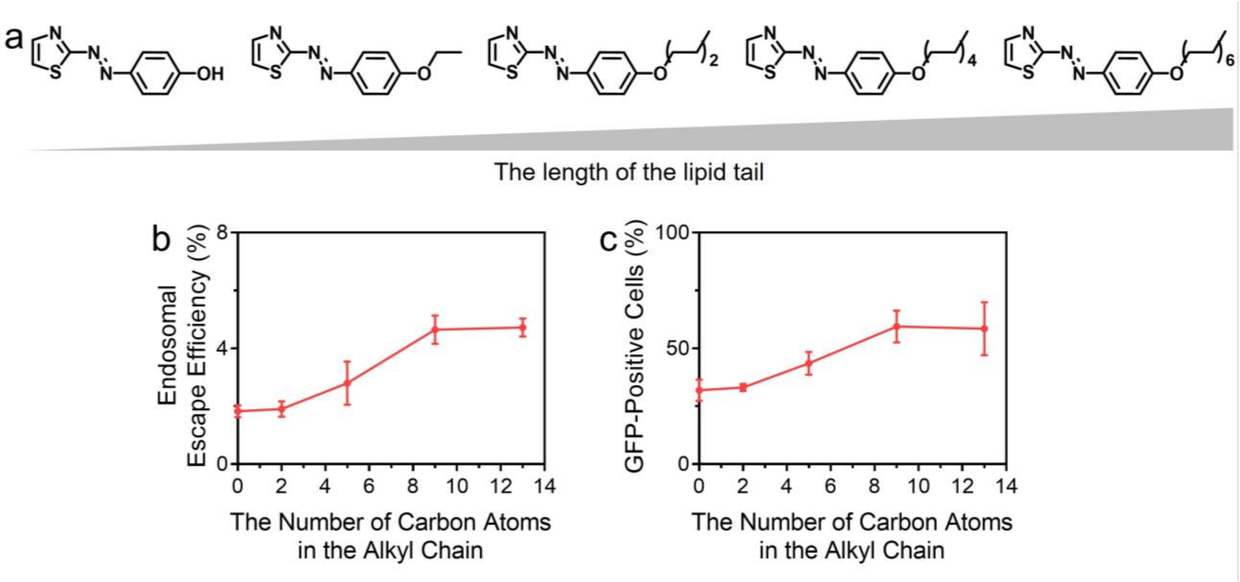

Fig. S5. Optimization of alkyl chain length of PAT lipid. a) Chemical structure of PAT lipids with different alkyl chain lengths. b) The correlation between endosomal escape efficiency and alkyl tail chain length of PAT lipid. c) The correlation between mGFP expression and alkyl tail chain length of PAT lipid. Data are presented as mean ± standard deviation (s.d.) from *n* biologically independent samples (*n* = 3).

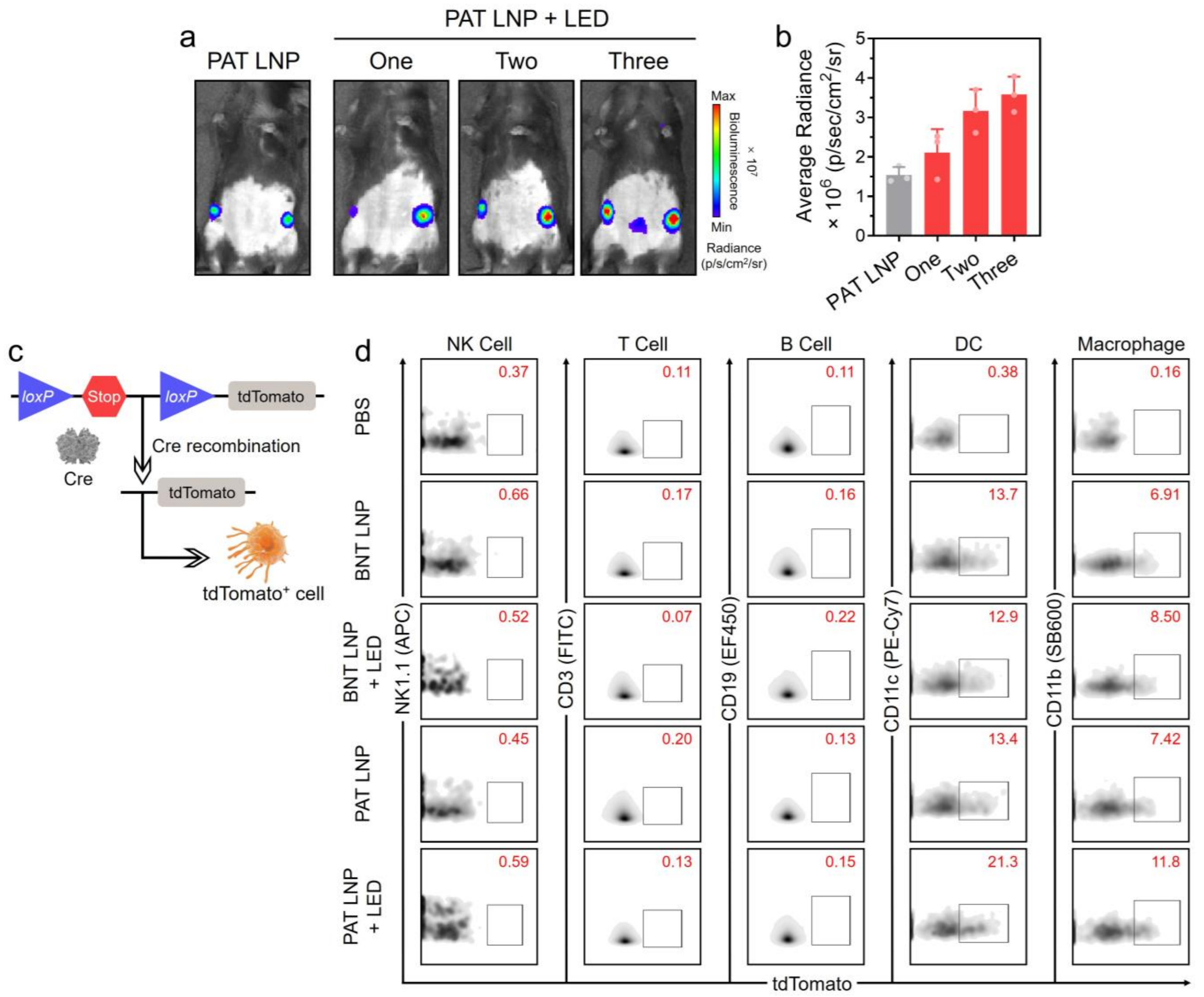

Fig. S6. Delivery of mCre to lymph nodes (LNs) in Ai14 mice using PAT LNPs. a) Representative bioluminescence images of the mice after SC injection of mLuc-loaded PAT LNPs (mRNA, 5 µg per mouse) and received different irradiations measured by IVIS imaging system. b) bioluminescence intensity within inguinal LNs. c) Mechanism of Cre-mediated gene recombination in Ai14 reporter mice. The gene encoding tdTomato is blocked by a stop gene (red) between two *loxP* segments (blue). When mCre is delivered to the cytoplasm and expressed, the *loxP* gene will be cut. This activates the tdTomato gene, leading to the expression of tdTomato in the cells. d) Percentage of tdTomato-positive (tdTomato^{+}) cells in different types of immune cells after administration of mCre-loaded LNPs. Naked mCre is employed as control.

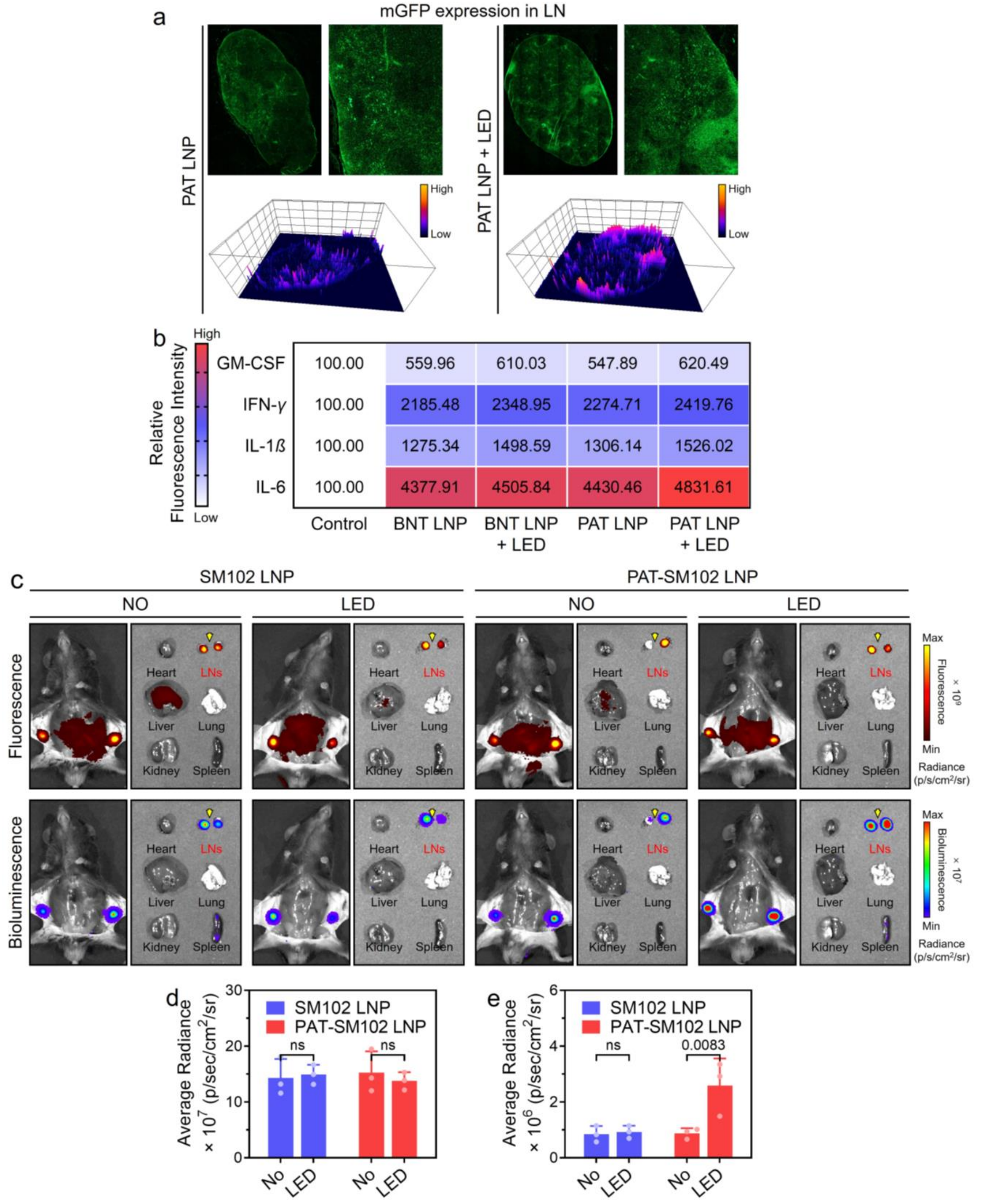

Fig. S7. Evaluation of cytokines in LNs. a) Fluorescent images of mGFP expression in the whole LNs and the intensity analysis showed in Fig. 3F. b) Inflammatory cytokine levels in LNs after different treatments. c) Representative fluorescence (top) and bioluminescence (bottom) images of the mice after SC injection of DiR-labeled mLuc-loaded LNPs (mRNA, 5 µg per mouse) measured by IVIS imaging system. d) Quantitative analysis of the fluorescence intensity and e) bioluminescence intensity within inguinal LNs.

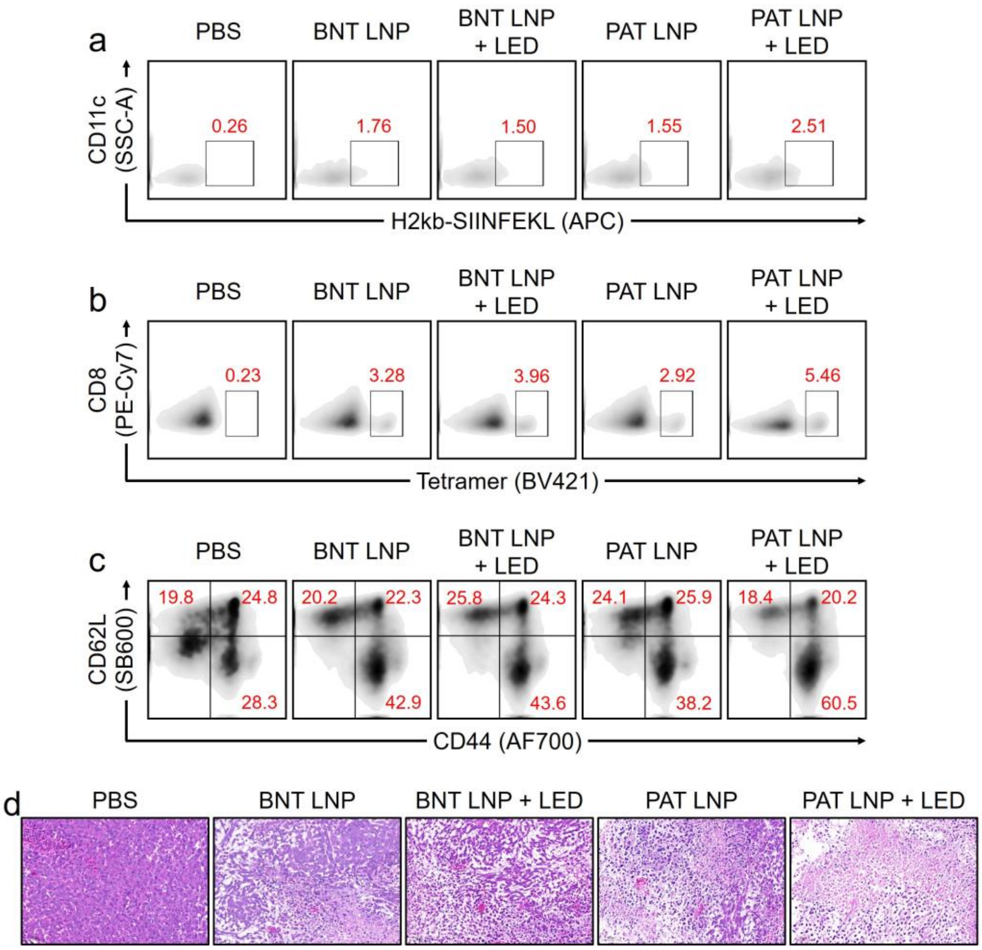

Fig. S8. Prevention of the melanoma growth in a mouse model. a) Flow cytometry analysis of the population of DC cells that express H2kb-SIINFEKL (gated on $Ly6G^{-}CD11c^{+}$ cells) in inguinal LNs. b) Flow cytometry analysis of the population of $CD8^{+}CD3^{+}$ T cells that bear T cell receptors binding to H2Kb OVA tetramer-SIINFEKL in inguinal LNs. c) Flow cytometry analysis of the population $CD62L^{low}CD44^{high}$ T cells (gated on $CD8^{+}CD3^{+}$ cells) in inguinal LNs. d) Hematoxylin and eosin (H&E) staining of tumor tissues aftter different treatments.

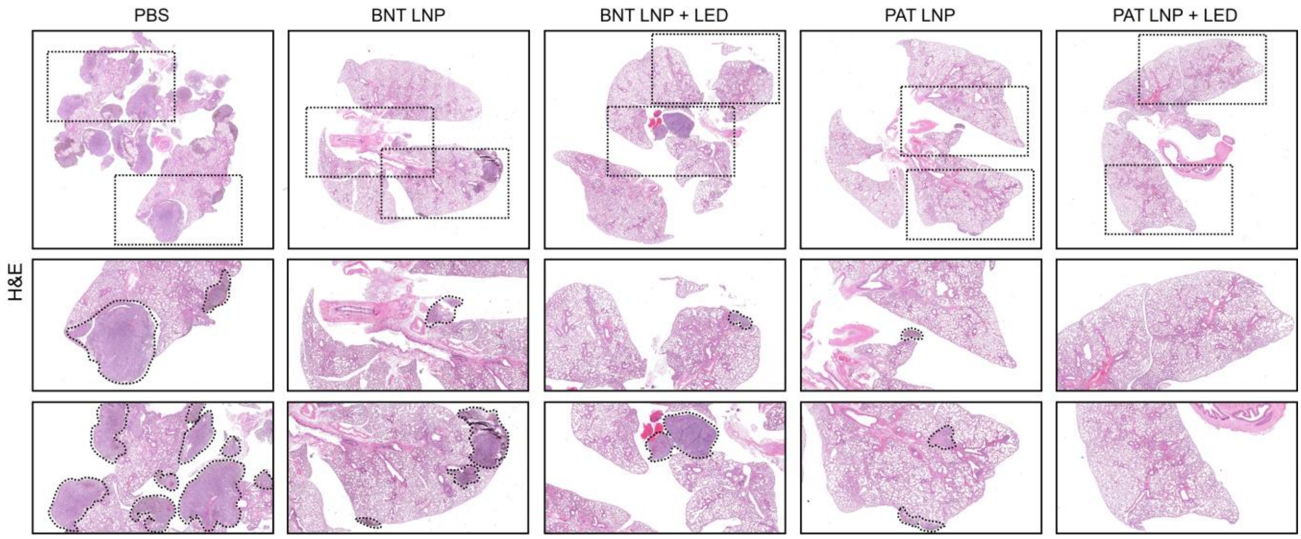

Fig. S9. Prevention of tumor metastasis to lungs. H&E staining images (including enlarged field of view) of the lung tissues showed in Fig. 4M**.**

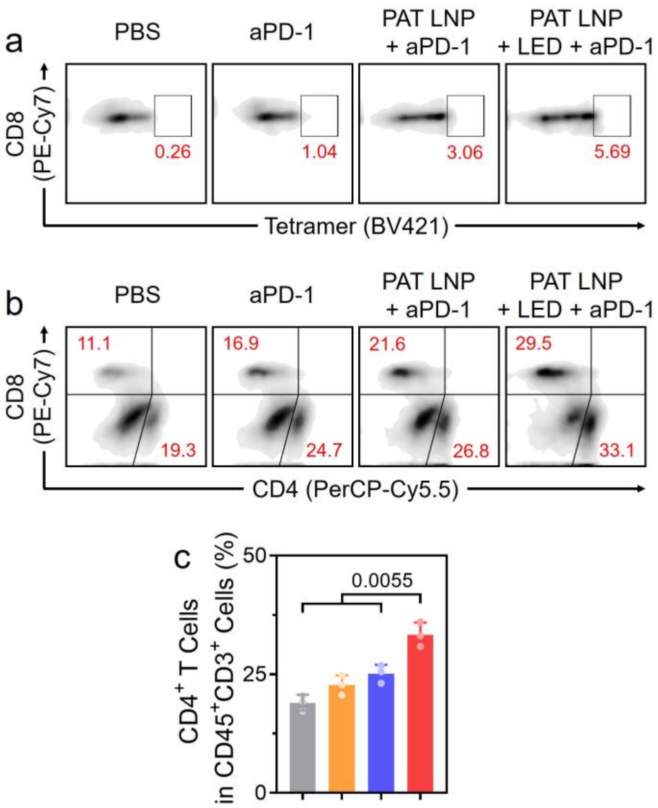

Fig. S10. Prevention of postoperative recurrence of glioblastoma. a) Flow cytometry analysis of the population of DC cells that express H2kb-SIINFEKL (gated on Ly6G$^-$CD11c$^+$ cells) in axillary LNs. b) Flow cytometry analysis of the population of CD8+ T cells (gated on CD3$^+$CD45$^+$ cells) in GL261-OVA-Luc tumors. c) Flow cytometry analysis of the population of CD4$^+$ T cells (gated on CD3$^+$CD45$^+$ cells) in GL261-OVA-Luc tumors. Data are presented as mean ± s.d. from *n* biologically independent samples (*n* = 3). Statistical significance was analyzed by one-way ANOVA with Tukey's multiple comparisons test for **c**.

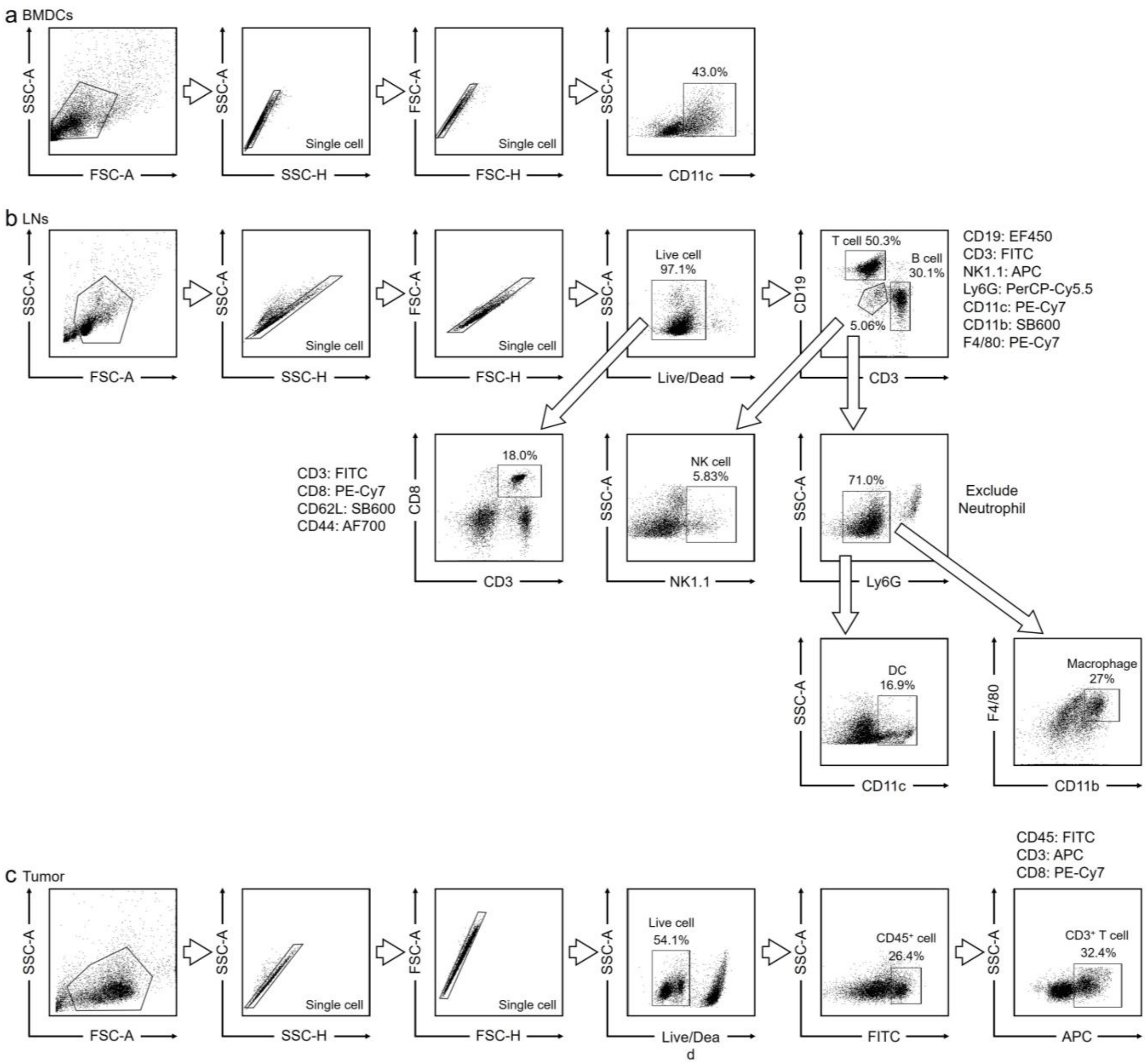

Fig. S11. Gating strategy for identification of lymphocytes.
